# Lithium isotope effects and magnetic interactions in flavin–ascorbyl radical pairs

**DOI:** 10.1371/journal.pone.0356617

**Published:** 2026-09-10

**Authors:** Amina Mouhamed, Jim Al-Khalili, Marco Sacchi

**Affiliations:** 1 Leverhulme Quantum Biology Doctoral Training Centre, University of Surrey, Guildford, United Kingdom; 2 School of Chemistry and Chemical Engineering, University of Surrey, Guildford, United Kingdom; 3 School of Mathematics and Physics, University of Surrey, Guildford, United Kingdom; Monash University, AUSTRALIA

## Abstract

For decades, lithium carbonate has been a cornerstone treatment for bipolar disorder and related conditions, yet its mechanism of action remains poorly understood. Previous studies have suggested that the two stable isotopes, ^6^Li and ^7^Li, with nuclear spins of 1 and 3/2, respectively, can produce distinct biological effects. Because nuclear spin can subtly alter the course of specific chemical reactions, these studies have motivated the exploration of whether lithium’s isotopes might act through a spin-dependent mechanism. Here, we investigate the Radical Pair Mechanism in the flavin–ascorbyl system using DFT and spin-dynamics calculations. The ascorbyl radical, derived from vitamin C, is both abundant in the brain and long-lived, making it a more credible partner for flavin. We propose that lithium’s nuclear spin modulates triplet yields in flavin–ascorbyl radical pairs, offering a quantum-based explanation for isotope-dependent effects. Whether these spin interactions play a clinical role remains to be determined, but the possibility is exciting and points toward a promising line of inquiry.

## Introduction

Lithium carbonate, introduced by John Cade in 1948, remains the most effective treatment for bipolar disorder, stabilising mood, preventing relapse, and reducing suicide risk [1,2]. It is the gold-standard therapy across acute and maintenance phases and continues to be widely prescribed despite its narrow therapeutic window [3]. Yet, more than seventy years later, the molecular basis of lithium’s therapeutic action remains unresolved.

Conventional explanations of lithium’s therapeutic action focus on its effects on neurotransmitter pathways and second messenger systems [4]. At the molecular level, lithium displays several unusual features. It has no dedicated transporters, yet can enter cells through Na^+^ channels and K^+^ channels [5]. And although it lacks specific binding sites, it can compete with Mg^2+^ in key biochemical processes. For example, ATP normally binds Mg^2+^ at defined phosphate groups, but Li^+^ can substitute for Mg^2+^ in these complexes, as shown by DFT calculations and solid-state NMR studies [1]. The geometry of the enzyme binding site appears to govern how effectively Li^+^ competes with Mg^2+^, a leading hypothesis for lithium’s biochemical action [6]. Within pharmacological models, the lithium used in treatment is not isotopically pure but reflects its natural abundance, consisting of approximately 7.6% ${\;}^{6}Li$ and 92.4% ${\;}^{7}Li$. [7].

Both isotopes contain three protons and three electrons but differ by one neutron. They therefore differ by about 14% in mass and by their nuclear spin, giving them distinct masses (6.015 g/mol for ^6^Li and 7.016 g/mol for ^7^Li) and different nuclear spins (*I* = 1 for ^6^Li and *I* = 3/2 for ^7^Li) [8]. Each of these differences could cause the isotopes to behave slightly differently in the body, although this was not considered in routine clinical use [9].

Consistent with this possibility, in animal studies, ^6^Li appears to produce stronger behavioural effects than ^7^Li, particularly in reducing ketamine-induced hyperactivity and spontaneous motor activity in rats [10,11], although these differences may diminish over time. Natural isotope variations in sheep suggest that ^6^Li is preferentially absorbed in the intestine and enters red blood cells (erythrocytes) more rapidly than ^7^Li [12]. At the cellular level, the evidence is less consistent. Livingstone et al. reported that ^6^Li more effectively reduced sodium levels in erythrocytes than ^7^Li [13], suggesting a possible isotope effect on ion transport across cell membranes. However, no significant differences were found between the isotopes in neuronal toxicity, in glycogen synthase kinase 3 beta (GSK-3β, an enzyme involved in cell signalling and metabolism) phosphorylation, or in overall kinase activity [7]. Similarly, only minor variations were observed in sodium channel activation in neurons derived from induced pluripotent stem cells (iPSCs, adult cells reprogrammed to behave like stem cells), and no isotope effect was detected in SH-SY5Y neuroblastoma cells (a human cell line commonly used as a neuronal model). Adding to this picture, recent clinical data reveal that serum $\delta^{7}Li$ decreases by ~11‰ in bipolar disorder and ~5‰ in schizophrenia following lithium treatment, pointing to serum isotope fractionation as a new biomarker [14]. Taken together, these findings indicate that isotope-dependent effects of lithium are inconsistent across systems and remain difficult to account for within standard biochemical models. Either differences in mass, which influence reaction rates, or differences in nuclear spin, which produce spin-dependent effects, can lead to isotope differences.

However, a growing body of work has begun to view nuclear spin as a plausible contributor, motivating further exploration of spin-dependent mechanisms [2,15–17]. Such mechanisms have been invoked to account for the anaesthetic potency of xenon [17], the behavioural effects of lithium isotopes [2], magnetic-field modulation of NMDA receptor activity [18], microtubule reorganisation [19], and ROS-mediated effects on neurogenesis [20] and planarian regeneration [21].

Most recently, Esmaeilpour et al. [22] reported the first observation of large and opposite effects of lithium isotopes on neuronal function using multielectrode array electrophysiology in rat brain tissue. The opposite effects of the two lithium isotopes on synaptic transmission, together with the rapid onset of the distinct electrophysiological response observed for ${\;}^{6}Li$, suggest that the underlying mechanism is more likely related to differences in nuclear spin than to mass-dependent effects.

### The radical pair mechanism

Among nuclear-spin–based explanations, the Radical Pair Mechanism (RPM) has emerged as a prominent candidate for magnetic isotope effects in biology [19,23–26]. The RPM provides a route by which nuclear magnetic interactions influence chemical reactions. To introduce the key elements: a radical is a molecule with an unpaired electron, and a radical pair (RP) forms when two such unpaired electrons end up on two nearby molecules. This can happen when a bond breaks homolytically (each atom takes one electron), when an electron is transferred between two closed-shell molecules, or when two radicals meet in solution [27]. Each unpaired electron carries an intrinsic quantum-mechanical property known as electron spin, which gives rise to a magnetic moment and determines the possible spin states of a radical pair. When two radicals are created, their electrons’ spins can couple in two ways: either a spin-0 singlet state (antiparallel spins) or a spin-1 triplet state (parallel spins). After formation, these two states can interconvert coherently, a process often referred to as singlet–triplet mixing or quantum beating. This interconversion is driven by hyperfine interactions between electron spins and nearby atomic nuclei, as well as by electron-electron coupling. This spin coupling can then shape the subsequent chemistry: singlet states tend to recombine into stable products, whereas triplet states favour radical separation or alternative reaction pathways. Because nuclear spins directly influence singlet–triplet mixing, replacing one isotope with another can alter reaction rates and yields whenever their nuclear spins differ [28]. Even subtle isotope-dependent changes in these reaction pathways could alter chemical signalling or redox processes, with potential consequences for broader physiological function [24,29].

In biology, one common site where RP are formed is the Cryptochrome (Cry) blue-light receptor, which generates the flavin radical($FADH^{•}$) during its photochemical cycle [24,27,30].

### The flavin–superoxide model

This line of reasoning underlies the framework proposed by Zadeh-Haghighi et al., who argued that lithium’s isotope effects on hyperactivity may arise from magnetic isotope effects mediated by the RPM [24]. Much of the theoretical framework used in the present work follows directly from their approach, including the choice of the experimental reference point; the main difference introduced here is the substitution of the radical partner.

In their model, the RP consists of a flavin semiquinone ($FADH^{•}$) and a superoxide anion ($O_{2}^{•-}$) interacting with a *Li*_+_ ion. Superoxide is part of the broader family of reactive oxygen species (ROS), which includes both radical species such as superoxide and non-radical signalling molecules such as hydrogen peroxide ($H_{2}O_{2}$). At low concentrations, ROS act as important redox-signalling agents, especially $H_{2}O_{2}$ and $O_{2}^{•-}$, whereas imbalances can lead to oxidative stress, a process associated with bipolar disorder [31]. Moreover, superoxide’s dominant isotope, ${\;}^{16}O$, has no nuclear spin, making the $[FADH^{•}$ $O_{2}^{•-}]$ pair a tempting system for examining spin-dependent dynamics. Once formed, this RP can either recombine in the singlet state to form $H_{2}O_{2}$ and oxidised flavin, or dissociate in the triplet state to release $O_{2}^{•-}$. Weak electromagnetic fields have been shown to modulate ROS production via Cry, influencing cellular outcomes in an exposure-dependent manner [32]. Because ^6^Li and ^7^Li differ in nuclear spin, the RPM predicts isotope-dependent shifts in singlet–triplet mixing and therefore in the final chemical yields. This prediction has been noted to align with the behavioural isotope ratios reported by Ettenberg *et al.* [10]. A follow-up study extended this idea to circadian biology, proposing that magnetic fields and lithium could modulate radical-pair dynamics in Cry and alter the circadian period in an isotope-dependent manner [15].

A major strength of the RPM is that it provides a clear theoretical basis for generating testable predictions. However, meaningful comparison with experiments requires models that reflect realistic biological conditions, even if this increases computational complexity [28]. This requirement poses a challenge for the superoxide-based proposal.

Superoxide relaxes on the nanosecond timescale, a consequence of its orbitally degenerate ground state (${\;}^{2}\Pi_{g}$) and strong spin-orbit interaction, which links the electron spin to the orientation of the molecule [33]. In solution, molecular rotation therefore leads to rapid loss of electron spin coherence, occurring on the same timescale as rotational motion. As a consequence, the lifetime of spin coherence in $O_{2}^{•-}$ is too short to support magnetic-field-driven singlet–triplet mixing or other coherent spin dynamics. Radical pairs that relax faster than 1 μs show minimal sensitivity to a 50 μT field. [34] This rapid relaxation also explains why liquid-phase Electron Paramagnetic Resonance (EPR)(a method for detecting species with unpaired electrons) has never yielded observable spectra for $O_{2}^{•-}$. It also clarifies why clear magnetic-field effects (MFE) only appear at fields many orders of magnitude stronger than 50 μT [35–37]. Meaningful magnetic sensitivity would require $O_{2}^{•-}$ to be held in a fixed, asymmetric environment that lifts the $\pi_{g}$ orbital degeneracy and restricts tumbling. Only under such conditions does relaxation slow, consistent with EPR signals observed in frozen solutions [38] and alkali-halide crystals [39]. In liquid environments, however, $O_{2}^{•-}$ relaxes far too quickly to serve as a viable radical-pair partner. A second possibility is Kattnig’s proposed radical-scavenging pathway in cryptochrome, although this idea remains untested experimentally [40]. A recent computational study by Deviers et al. shows that superoxide can, in principle, act as a viable partner if it transiently binds to specific positively charged sites in cryptochrome, where binding can slow its tumbling by up to three orders of magnitude and extend relaxation times into the magnetically sensitive regime. However, these favourable binding events were rare and highly configuration-dependent, suggesting that such a mechanism would require a high level of orchestration [41]. In sum, freely tumbling superoxide is unlikely to sustain coherent spin dynamics at low fields, although rare micro-environmental trapping may create pockets of viability.

In addition, molecular oxygen’s triplet ground state means that such radical pairs most likely begin in the triplet configuration, which may conflict with behavioural observations [25,28,42].

These limitations motivate the search for alternative reaction partners that satisfy both the quantum requirements for low-field sensitivity and the physiological conditions present in the brain.

### The ascorbyl radical

One promising candidate is the ascorbyl radical. It has already been introduced in the context of the brain as part of a radical triad proposed to explain the effects of hypomagnetic fields on neurogenesis [25]. Ascorbic acid, a common biological reductant, can reduce photoexcited flavins and tryptophan radicals through hydrogen atom or electron transfer [43]. The resulting ascorbyl radical has one relatively large hyperfine interaction of about 0.2 mT and several smaller ones in the range of 0.01–0.02 mT [42]. Compared with the flavin–superoxide system, a flavin–ascorbic acid radical pair is more likely to exhibit magnetic field effects (MFEs) *in vivo* [44,45]. Ascorbic acid (vitamin C) is abundant in neurons (≈10 mM) and is thought to help protect the brain from oxidative stress by neutralising reactive oxygen species (ROS) produced during regular brain activity. Its concentration is not uniform across the brain: the hypothalamus, for instance, appears to contain relatively high levels of vitamin C [46,47]. Within the hypothalamus, the suprachiasmatic nucleus (SCN) regulates circadian rhythms, with Crys believed to play a key role in coordinating these cellular oscillations [2]. Lithium may also act directly on the SCN [48], and disruptions in this circadian centre are often reported in mood disorders [49].

**Fig 1:**
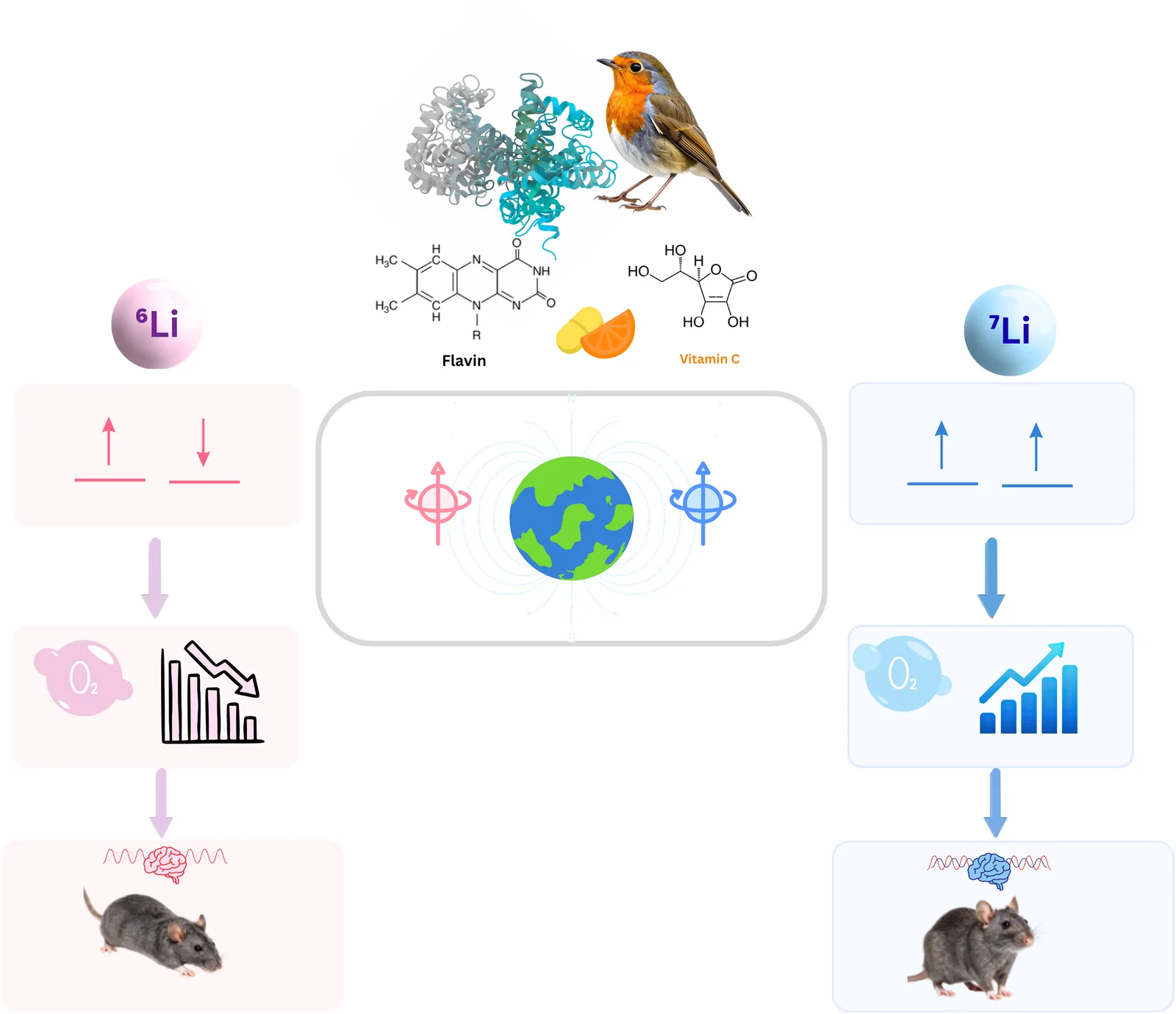
Lithium isotope effects on RP spin dynamics and behaviour. *Top:* a cryptochrome protein hosts a flavin–ascorbyl radical pair (${\text{FADH}}^{•}$–${\text{Asc}}^{•}$) formed by electron transfer from ascorbate. *Centre:* nuclear-spin-dependent singlet–triplet interconversion is governed by hyperfine interactions and the geomagnetic field. Singlet pairs recombine to regenerate antioxidant-active AscH^-^, whereas triplet pairs persist as the less effective ascorbyl radical (${\text{Asc}}^{•}$). *Left (*^6^*Li):* the spin-1 nucleus favours singlet character, enhancing recombination, preserving antioxidant capacity, lowering ROS levels, and attenuating hyperactivity. *Right (*^7^*Li):* the spin-3/2 nucleus increases triplet yield, allowing more radicals to escape as ${\text{Asc}}^{•}$, reducing antioxidant capacity, elevating ROS levels, and increasing hyperactivity, consistent with reported isotope-dependent behavioural differences in rats.

**Fig 2:**
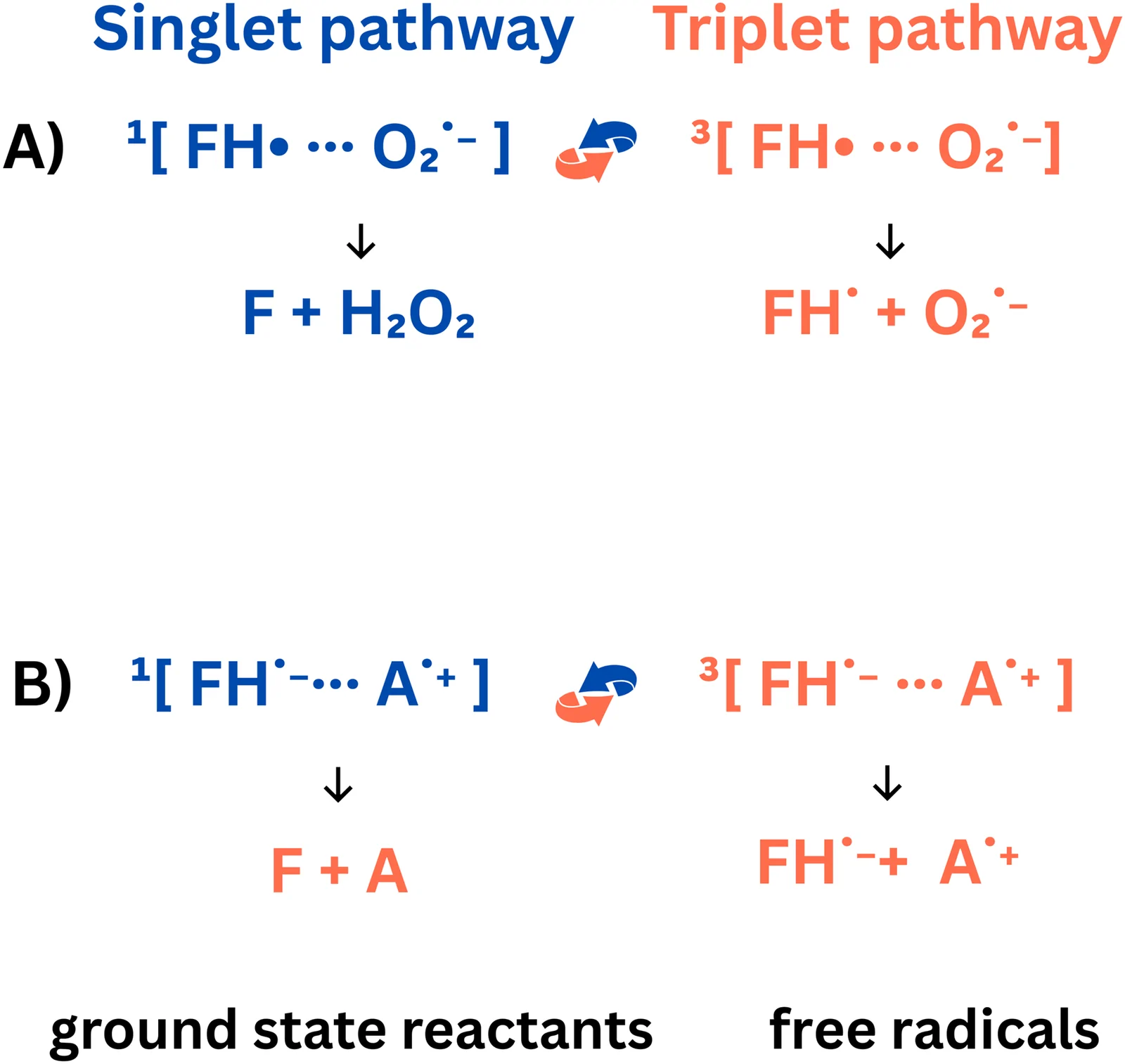
Proposed mechanisms. Panel A: Radical pair between flavin semiquinone ($FADH^{•}$) and superoxide ($O_{2}^{•-}$). Singlet recombination (blue) yields hydrogen peroxide (H${\;}_{2}{\text{O}}_{2}$) and oxidized flavin, whereas triplet dissociation (orange) releases superoxide. Panel B: Reaction scheme involving $FADH^{•}$ and ascorbic acid (A). The schematic illustrates ascorbate radical dynamics, adapted from the in vitro study by [44]. Singlet pathways (blue) lead to stable products (F+A), while triplet pathways (orange) generate reactive radical species ($FH^{•-}+A^{•+}$).

Here, we examine how lithium might influence the spin-dependent chemistry of such an RP. A general schematic view of the flavin–ascorbyl pair is shown in Fig 1, while Fig 2 illustrates the comparison with the superoxide-based model. Our approach has two components: (1) Density Functional Theory (DFT) calculations to obtain the hyperfine coupling constants of the radicals, and (2) Spin-dynamics simulations to compute the resulting triplet yields.

By modelling the electron density rather than the full many-electron wavefunction, DFT offers an efficient yet accurate description of molecular electronic structure. This approach simplifies the calculation while still providing high accuracy [50]. One of the key quantities obtained from these calculations are the hyperfine coupling constants (HFCC), which measure the interaction between the magnetic moment of the unpaired electron and nearby atomic nuclei [51].

These HFCCs serve as inputs to our spin-dynamics calculations, in which we model how the two lithium isotopes might influence triplet yield, i.e., the probability that an RP reacts from the triplet state rather than the singlet state. Changes in triplet yield reflect changes in the underlying spin-conversion dynamics, providing a link between lithium’s nuclear spin and isotope-dependent biological effects observed in behavioural studies by Ettenberg et al [10].

## Methods

The Methods section is organised into a DFT component (HFCC calculations) and a spin-dynamics component (triplet-yield calculations).

### DFT calculation of HFCC

HFCCs for both radicals were calculated using DFT as implemented in the ORCA 6 software package [52], which provides reliable methods for computing magnetic parameters such as g-tensors and HFCs [53].

A two-step procedure was followed, comprising geometry optimisation followed by an HFCC calculation. For consistency, the same dielectric environments were used for both steps. To assess solvent effects, computations were performed in two dielectric environments: a low-polarity medium (ε=2) and water (ε=80). Solvent effects were modelled using the Conductor-like Polarizable Continuum Model (CPCM), which approximates the solvent as a polarizable continuum characterised primarily by its dielectric constant and refractive index. The low-polarity calculations allow for direct comparison with previous work on superoxide. For the aqueous environment, calculations were performed using both CPCM and the Solvation Model based on Density (SMD). While CPCM accounts primarily for electrostatic solvent effects, SMD also includes non-electrostatic contributions such as dispersion, cavitation, and solvent structure. [54] HFCCs obtained with SMD showed improved agreement with EPR data for both flavin and ascorbyl radicals. [55]

Geometries were optimised using the B3LYP functional with the 6–311++G(d,p) basis set, D4 dispersion corrections, and the appropriate solvation model (CPCM or SMD). Optimised structures were validated by comparison with available experimental bond lengths and angles [56]. We note that agreement with experimental HFCCs may partly result from error compensation and the exclusion of effects such as explicit solvation, conformational flexibility, and vibrational averaging.

Assuming random orientation of the proteins, only isotropic Fermi contact terms were included in the hyperfine interaction calculations. As no experimental HFCC data exist for the full [Li–Asc] complex, the method was first validated using the ascorbyl radical. Isotropic HFCCs calculated with different density functionals are summarised in Table A (Supporting Information, S1 Text in S1 File). The M06 functional combined with the def2-QZVPP basis set, D4 dispersion corrections, and SMD solvation provided the closest agreement with experimental values [47,55] and was therefore used for all subsequent [Li–Asc] calculations. M06 is a global hybrid meta-GGA functional widely used in studies of organic radicals [57].

The same functional and basis set were used for both radicals to keep the calculations consistent.

**Fig 3:**
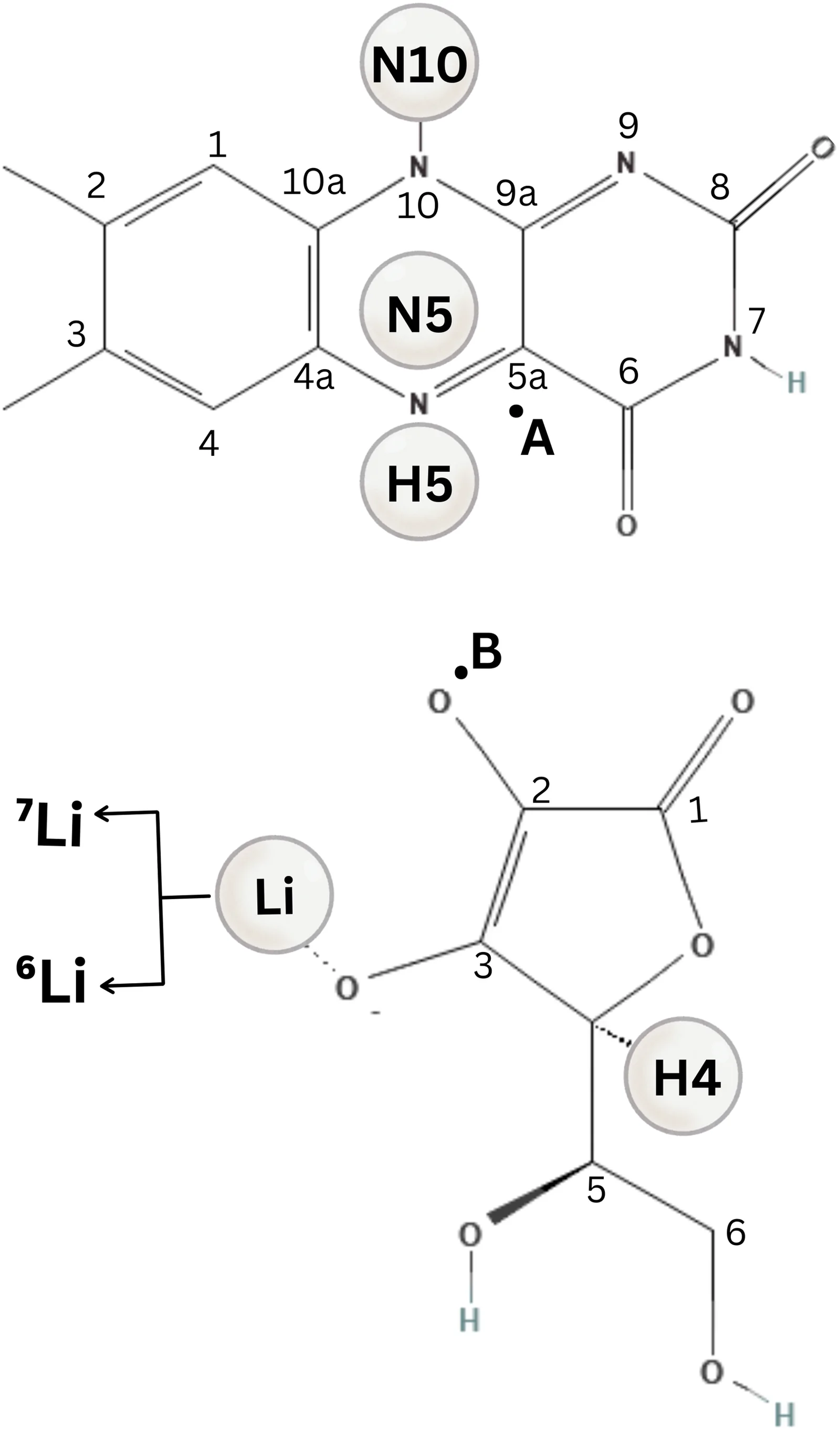
Schematic of the flavin–lithium ascorbyl radical pair. Unpaired electrons are indicated by dots: Electron A is located on flavin (near N5, top), and Electron B on ascorbyl (near H4, bottom). The grey spheres mark the magnetic nuclei that interact with the unpaired electrons through hyperfine coupling. In the isoalloxazine ring of flavin, the key magnetic nuclei (N5, N10, and H5) are highlighted. The interaction can be influenced by the bound lithium isotope (${\;}^{6}Li$ or ${\;}^{7}Li$), as shown by the arrows. Relative positions are illustrative and do not represent an optimised geometry.

Because the ${\text{FADH}}^{•}$ radical is relatively large, we used a truncated ${\text{FMNH}}^{•}$ model for the hyperfine calculations. In contrast to ${\text{FMNH}}^{•}$, the full ${\text{FADH}}^{•}$ cofactor includes an additional adenine moiety attached to the ribityl–phosphate tail. This adenine unit was removed to reduce computational cost. This truncation is well-precedented in the literature [2,20,42,58], and our calculated HFCCs show good agreement with available EPR data, confirming its suitability here [59]. The unpaired electron is localised on the isoalloxazine ring, which dominates the hyperfine interactions (see Table C in S1 File). This ring contains three key magnetic nuclei: N5, N10, and H5, shown in Fig 3.

Moreover, in the neutral ${\text{FADH}}^{•}$ radical, Zadeh-Haghighi et al. ()(2021) [2] reported that the unpaired electron in Cry couples most strongly to the N5 nucleus, which has the largest isotropic hyperfine coupling constant. Because H5 also makes a noticeable contribution, we performed additional calculations to evaluate its effect (see Supporting Information, S2 Text in S1 File).

The final isotropic HFCCs for ${\text{Asc}}^{•}$ and ${\text{FADH}}^{•}$ in each environment were then used as input parameters in the spin Hamiltonian (Eq. 2) to calculate the triplet yields. Because the HFCs of the two lithium isotopes scale in proportion to their magnetogyric ratios, it is sufficient to perform the DFT calculation for only one isotope and obtain the other by simple scaling.

### Triplet yield ratio calculations

To compute the triplet yields, we implemented the spin-dynamics model developed by Hore [58], which describes the time evolution of radical-pair spin states under hyperfine interactions and the external magnetic field. In this framework, the radical pair is represented by a spin Hamiltonian containing the Zeeman terms for each electron and the relevant hyperfine couplings. The singlet–triplet interconversion is obtained by propagating the density matrix in time. A mathematical description of this formalism is presented in the Model Section, with full details provided in Supporting Information S3 Text in S1 File.

The complete Python code used for these calculations is available in Supporting Information S4 text in S1 File. Our implementation was validated against a simplified three-spin system, reproducing the benchmark results reported by Rodgers [30]. Triplet yields for all systems in this study were then computed in Python using this validated model.

## Model

We study a RP formed between flavin semiquinone (${FADH}^{•}$) and the ascorbyl radical (${Asc}^{•}$), as illustrated in Fig 3. Although the figure depicts the general system, several possible pathways may lead to its formation. The first involves random encounter pairs, which arise when diffusing ascorbyl and flavin radicals meet in solution. [25,44]. The second involves geminate pairs, arising from photoinduced or enzymatic electron transfer between ascorbic acid and flavin cofactors, producing spin-correlated radicals that retain their initial singlet or triplet configurations. Although such mechanisms have been demonstrated experimentally *in vitro* [44], their relevance in neuronal tissue remains to be established.

The flavin radical itself may occur in several biochemical contexts. It can be protein-bound, for example, as a semiquinone cofactor within flavoproteins such as NADPH oxidases, which generate ROS in cells [20], or it may exist freely in solution, interacting with other radicals independently of a protein framework. [25]

Within this flavin-ascorbyl RP, unpaired electron A resides on flavin and unpaired electron B on ascorbyl. Because ${Asc}^{•}$ carries a negative charge, it can transiently associate with a lithium ion via electrostatic coordination to its oxygen atoms, forming a lithium–ascorbyl complex [LiAsc]. The coordination site for Li^+^ is near the negatively charged third oxygen [60]. In this position, the lithium nucleus lies sufficiently close to magnetically couple with electron B.

As mentioned before, Li has two stable isotopes, ${\;}^{6}Li$ (*I* = 1) and ${\;}^{7}Li$ ($I=\frac{3}{2}$). Their different nuclear spins and magnetic moments could modify the hyperfine interaction with electron B. This, in turn, could affect the rate of singlet-triplet interconversion in the RP $\left[{FADH}^{•}\cdots {Asc}^{•}\right]$. As a result, the singlet-triplet balance, and consequently the reaction yields, depend on which isotope is present.

### Mathematical formulation

We adopted the RPM framework described by Haghighi et al. [2], while using Hore’s spin-dynamics formalism [58]. Details of the triplet yield derivation are provided in Supporting Information S3 Text in S1 File.

Briefly, the Hamiltonian ($\hat{H}$) describes how the two unpaired electrons interact with nearby nuclei and with an external magnetic field. It can be written as the sum of a Zeeman term and a hyperfine term:


$$\begin{array}{c} \hat{H}={\hat{H}}_{Zeeman}+{\hat{H}}_{Hyperfine}. \end{array}$$ (1)

The Zeeman term describes how each electron spin responds to the external magnetic field, while the hyperfine term captures the interactions between each electron and the nuclei in its local environment. For simplicity, we neglect inter-radical interactions. Quantum mechanical simulations show that significant changes in product yields can still occur at separations of about 2.0±0.2 nm, where exchange and dipolar contributions may partially cancel. [24,61,62] Under these conditions, and in biological environments where at least one radical is mobile, the dipolar interaction averages to nearly zero. [24,25]

For the biological RP shown in Fig 3, we retain only the dominant hyperfine interactions (N5, H4, and Li). The initial spin state was assumed to be a pure singlet. By convention in spin chemistry, $\hat{𝐒}$ denotes electron spin operators, while $\hat{𝐈}$ refers to nuclear spin operators. In the isotropic limit, this Hamiltonian is written as the sum of contributions from radical A and radical B:


$$\begin{array}{c} {\hat{H}}_{Flavin\text{-}Ascorbyl}=(\omega {\hat{S}}_{A_{z}}+a_{N5}\;{\hat{𝐒}}_{A}\cdot {\hat{𝐈}}_{N})+(\omega {\hat{S}}_{B_{z}}+a_{H4}\;{\hat{𝐒}}_{B}\cdot {\hat{𝐈}}_{H}+a_{Li}\;{\hat{𝐒}}_{B}\cdot {\hat{𝐈}}_{Li}) \end{array}$$ (2)

Here, ${\hat{𝐒}}_{A}$ and ${\hat{𝐒}}_{B}$ are the electron spin operators of radicals A and B, and ${\hat{𝐈}}_{A_{i}}$ and ${\hat{𝐈}}_{B_{i}}$ are the nuclear spin operators of the *i*th nuclei coupled to each radical through the hyperfine constants $a_{A_{i}}$ and $a_{B_{i}}$. The first and third terms ($\omega {\hat{S}}_{A_{z}}$ and $\omega {\hat{S}}_{B_{z}}$) describe the Zeeman interactions, while the other terms account for the hyperfine interactions. The parameter ω represents the electron Larmor frequency of the electrons due to the Zeeman effect. Mathematically, this frequency is given by $\omega =\gamma_{e}B$, where $\gamma_{e}$ is the electron gyromagnetic ratio and *B* is the applied magnetic field strength.

The model includes three HFCCs: $a_{N5}$, $a_{H4}$, and $a_{Li}$. The lithium term $a_{Li}$ describes the hyperfine interaction of the lithium ion bound to the ascorbyl radical and applies to either isotope, ${\;}^{7}Li$ (*I* = 3/2) or ${\;}^{6}Li$ (*I* = 1). In this notation, $a_{N5}$ refers to the coupling with the N5 nucleus of ${\text{FADH}}^{•}$, $a_{H4}$ to the dominant hydrogen in the ascorbyl radical. All HFCCs were taken from the DFT calculations described in the *Methods* section.

The time evolution of the spin system is described by the Liouville-von Neumann equation, which governs how the radical pair’s spin state changes with time. The eigenvalues and eigenvectors of the Hamiltonian are then used to calculate the triplet yield ($\Phi_{T}$), representing the probability that the radical pair ends up in the triplet state after recombination. A full derivation of Eq. 3 [58], including intermediate steps and assumptions, is provided in the Supporting Information S3 Text in S1 File. For times much longer than the radical pair lifetime, $\Phi_{T}$ can be expressed as


$$\begin{array}{c} \Phi_{T}=\frac{3(k+r)+k}{4(k+r)}-\frac{1}{M}\sum_{m,n=1}^{4M}\frac{{\left|\langle m|{\hat{P}}^{S}|n\rangle \right|}^{2}k(k+r)}{(k+r{)}^{2}+(\omega_{m}-\omega_{n}{)}^{2}}. \end{array}$$ (3)

The density matrix formalism was used to describe the time evolution of the radical pair. Here, $M=\prod_{j}(2I_{j}+1)$ is the total number of nuclear spin configurations, where $I_{j}$ is the nuclear spin quantum number of nucleus *j* (e.g., $I=\frac{1}{2}$ for ${\;}^{1}H$, *I* = 1 for ${\;}^{14}N$, etc.). The operator ${\hat{1}}_{M}$ denotes the identity in the nuclear spin subspace. The factor $\frac{1}{M}$ provides normalisation over all nuclear spin states. The operator ${\hat{P}}^{S}$ denotes the singlet projection operator, while |m⟩ and |n⟩ are eigenstates of the Hamiltonian ${\hat{H}}_{Flavin\text{-}Ascorbyl}$ with corresponding eigenenergies $\omega_{m}$ and $\omega_{n}$, respectively.

The parameter *k* is the recombination rate constant (s^-1^), which sets the rate at which the radical pair forms stable products. To keep the model straightforward, both reaction pathways were assigned the same first-order rate constant k, such that $k=k_{S}=k_{T}$ [63]. The parameter *r* is the spin relaxation rate constant (s^-1^), describing the loss of spin coherence over time. Both *k* and *r* are key parameters as they directly influence our analysis. Spin relaxation and chemical reaction terms were initially excluded and later incorporated following the approach of Bagryansky et al [64].

### Behavioural measurements reported by Ettenberg et al

The present work is computational; all behavioural data are drawn from previously published measurements reported by Ettenberg *et al.* [10], which we summarise below.

Lithium isotope effects were assessed using a ketamine-induced hyperactivity model of mania. Male rats received chronic dietary treatment with either ^6^Li, ^7^Li, or naturally abundant lithium chloride; control animals received sodium chloride. After 30 days, the rats were injected with ketamine (25 mg/kg) to induce hyperactivity, and locomotor activity was measured over 60-minute sessions. All lithium treatments reduced ketamine-induced hyperactivity relative to controls, but ^6^Li produced a significantly stronger and more sustained suppression of activity than ^7^Li or Li${\;}_{N}$(naturally occurring lithium). These results provide a quantitative behavioural benchmark for comparison and to motivate the use of the experimental ratio *TDr* in our RPM analysis.

Following the notation introduced by Zadeh-Haghighi *et al.* [2], we define the total travelled distance ratio as:


$$\begin{array}{c} TDr=\frac{TD_{7}}{TD_{6}} \end{array}$$ (4)

where *TD*_7_ and *TD*_6_ are the mean total distances (in cm) travelled by rats treated with ^7^Li and ^6^Li, respectively. In their study, the reported values were


$$\begin{array}{c} TD_{6}=20112\pm 929\text{ cm},\;TD_{7}=25630\pm 983\text{ cm},\;\text{giving}\;TDr=1.30\pm 0.08. \end{array}$$

The model-predicted ratio, *TYr*, is defined as:


$$\begin{array}{c} TYr=\frac{TY_{{\;}^{7}}}{TY_{{\;}^{6}}}=\frac{\Phi_{T}({\;}^{7}Li)}{\Phi_{T}({\;}^{6}Li)}. \end{array}$$ (5)

Here, $TY_{{\;}^{7}}=\Phi_{T}({\;}^{7}Li)$ and $TY_{{\;}^{6}}=\Phi_{T}({\;}^{6}Li)$ denote the triplet yields for the ^7^Li and ^6^Li systems, respectively, calculated using Eq. 3. The *TYr* notation follows previous work [10] but is fully equivalent to the ratio of the two $\Phi_{T}$ values.

Finally, the absolute difference


$$\begin{array}{c} \left|TDr-TYr\right| \end{array}$$ (6)

then measures how closely the RPM model matches the experimental isotope ratio, following the metric proposed in earlier work [2], and is used in the analysis shown in Fig 5. This comparison should be viewed as a coarse approximation, since the triplet-yield ratios do not capture the biochemical and physiological steps that lie between radical-pair spin dynamics and the behavioural distances measured by Ettenberg et al.

## Results

### DFT results

DFT calculations for the [Li-Asc] complex were used to assess environmental effects on the hyperfine couplings. Benchmark results are reported in Table B (Supporting Information, S1 file in S1 File))

Table 1 shows a clear dependence on the dielectric constant. For straightforward comparison with previous work on superoxide [2], the low-polarity environment (ε=2) was used in subsequent analyses, corresponding to the HFCCs listed in Table 1.

**Table 1:** Hyperfine coupling constants (*a*, in mT) calculated for the lithium–ascorbyl complex ([Li–Asc]) in different dielectric environments.

Environment	$a_{H4}$	$a_{Li7}$	$a_{Li6}$
Aqueous (ε=80)	0.288	0.1723	0.065
Low-polarity (ε=2)	0.423	0.224	0.083

### Isotope effects in the ascorbyl-based system

We first examined whether the ascorbyl radical, when paired with flavin in the $\left[{\text{FADH}}^{•},...,{\text{Asc}}^{•}\right]$ system, can produce a lithium isotope effect under biologically relevant conditions.

For that, we used the Hamiltonian in Eq. 2 with the HFCCs listed in the second row of Table 1. These values were then inserted into the Hamiltonian to calculate the triplet yields using Eq. 3. In this model, the unpaired electron in ${\text{FADH}}^{•}$ is assumed to couple only to the isoalloxazine N5 nucleus, consistent with previous studies.[15,42] Simulations were performed within a simplified RP framework using biologically relevant parameters: recombination rate constants *k* in the range $\left[10^{6}-10^{9},s^{-1}\right]$ and an ascorbyl relaxation rate constant fixed at $r=10^{6}s^{-1}$ [25,44].

**Fig 4:**
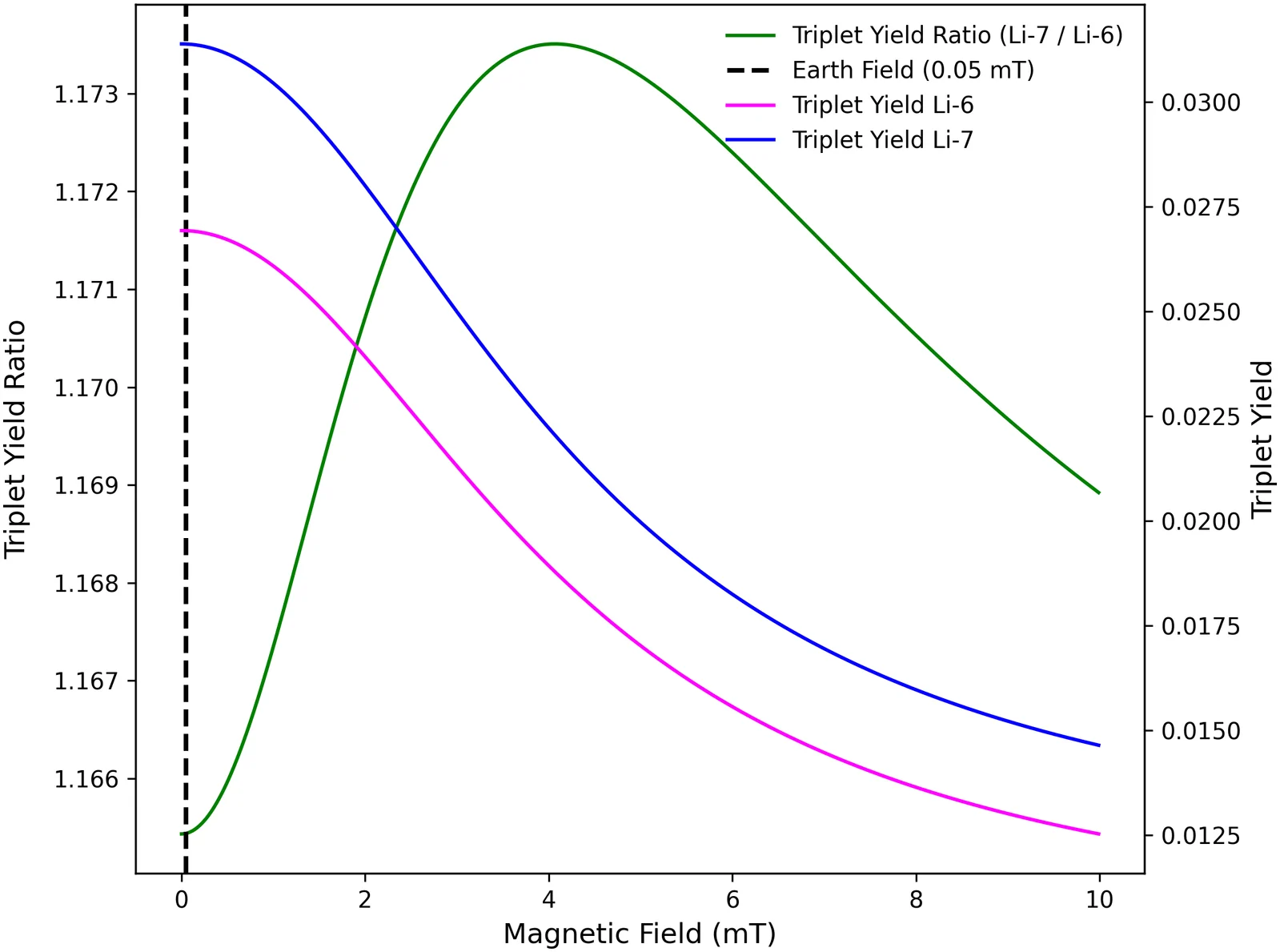
Magnetic-field dependence of triplet yields. The green solid line denotes the triplet yield ratio (${\;}^{7}Li/{\;}^{6}Li$), while the magenta and blue curves represent the absolute triplet yields for ${\;}^{6}Li$ and ${\;}^{7}Li$, respectively, as a function of the applied magnetic field strength. The vertical dashed black line indicates the Earth’s magnetic field (0.05mT). The hyperfine coupling parameters are: $a_{N5}=0.606\;mT$, $a_{Li6}=0.083\;mT$, $a_{Li7}=0.224\;mT$, and $a_{H4}=0.423\;mT$ (corresponding to the dominant hydrogen of the ascorbyl radical). Reaction and relaxation rate constants are $k=7\times 10^{8}\;s^{-1}$ and $r=10^{6}\;s^{-1}$, respectively. The value of *k* was chosen to remain within a realistic biological range and to keep the triplet ratio visible in the simulations. The relaxation rate *r* reflects the typical behaviour of the ascorbyl radical.

The results shown in Fig 4 reveal a clear isotope effect. At Earth’s magnetic field strength (~50 μT) and with realistic kinetic parameters, the calculated isotope ratio was 1.16, within 11% of the reported experimental value of 1.3. This ratio reaches a maximum of approximately 1.174 at 3–4 mT, providing a potential prediction for future experimental validation. Moreover, when hyperfine couplings were recalculated under aqueous conditions (ε=80), both the lithium–ascorbyl and flavin hyperfine constants decreased, yet the isotope ratio remained essentially unchanged (≈1.2).

The individual isotope yields (magenta and blue curves) exhibit RP behaviour: they peak in the 0−2 mT range and decline progressively with increasing magnetic field strength. However, the absolute yields are small (~0.026−0.031), raising questions about whether such differences could be experimentally distinguished above noise. Interestingly, while the magnetic field alters the absolute yields, it has little influence on the magnitude of the isotope effect, which remains nearly the same throughout.

The ascorbyl radical contains an additional strongly coupled hydrogen ($HF_{H4}=0.423\;mT$), which reduces its magnetic field sensitivity compared with superoxide, where such coupling is absent [25,42]. Despite this reduced sensitivity, our results suggest that lithium isotopic effects may still arise in the ascorbyl-based radical pair, implying that lithium could influence spin dynamics even in the presence of strong internal hyperfine fields.

### Direct comparison: Ascorbyl vs. superoxide systems

Earlier theoretical work has reported that a lithium isotope effect in flavin-superoxide radical pairs, with triplet–yield ratios of up to 1.17 at $k=4\times 10^{7}\;s^{-1}$ [2]. To place these results within a consistent modelling framework and to compare them directly with the flavin-ascorbyl system, we re-evaluated this ratio using a single parameter set for both radicals, including relaxation times representative of superoxide and ascorbyl.

The two radicals differ mainly in their relaxation rates: superoxide relaxes very quickly ($r=2\times 10^{8}\;s^{-1}$,4.9 ns), [25] whereas ascorbyl relaxes more slowly ($r=10^{6}\;s^{-1}$, microsecond scale). Under identical RPM conditions, we compared the two systems across a range of recombination rates (*k*) to assess how these relaxation rate constants affect magnetic sensitivity and the resulting lithium isotope effect. A direct comparison of the two systems is summarised in Table 2, which lists the isotopic triplet–yield ratios evaluated at the geomagnetic field across the same range of recombination rates.

**Table 2:** Isotope effect ratios evaluated at the geomagnetic field. Dimensionless ratios for flavin–superoxide and flavin–ascorbyl radical pairs calculated at the Earth’s magnetic field (50 µT) for different reaction rate constants *k*. The relaxation rate constants used were $r=2\times 10^{8}\;s^{-1}$ for superoxide (4.9 ns) and $r=10^{6}\;s^{-1}$ for ascorbyl (microsecond scale). Corresponding yield curves are shown in S5 File in S1 File.

*k* (s^-1^)	Flavin–Superoxide	Flavin–Ascorbyl
$4\times 10^{7}$	0.973	1.005
$4\times 10^{8}$	1.015	1.182
$1\times 10^{9}$	1.011	1.221

At $k=4\times 10^{7}$ s^-1^, neither system shows a noticeable isotope effect, with yield ratios remaining close to 1.0 across all magnetic field strengths. As *k* increases to $4\times 10^{8}$ s^-1^ and 10^9^ s^-1^, an isotope effect appears in the ascorbyl system, while superoxide remains largely insensitive. Specifically, at $k=4\times 10^{8}$ s^-1^, ascorbyl yield ratios slightly exceed unity, whereas superoxide stays near 1.0. The contrast becomes more pronounced at *k* = 10^9^ s^-1^, where the ascorbyl system shows its strongest isotope effect, with yield ratios reaching 1.221, while superoxide still exhibits minimal response to magnetic field changes.

### Identifying biologically relevant hot-spots in parameter space

**Fig 5:**
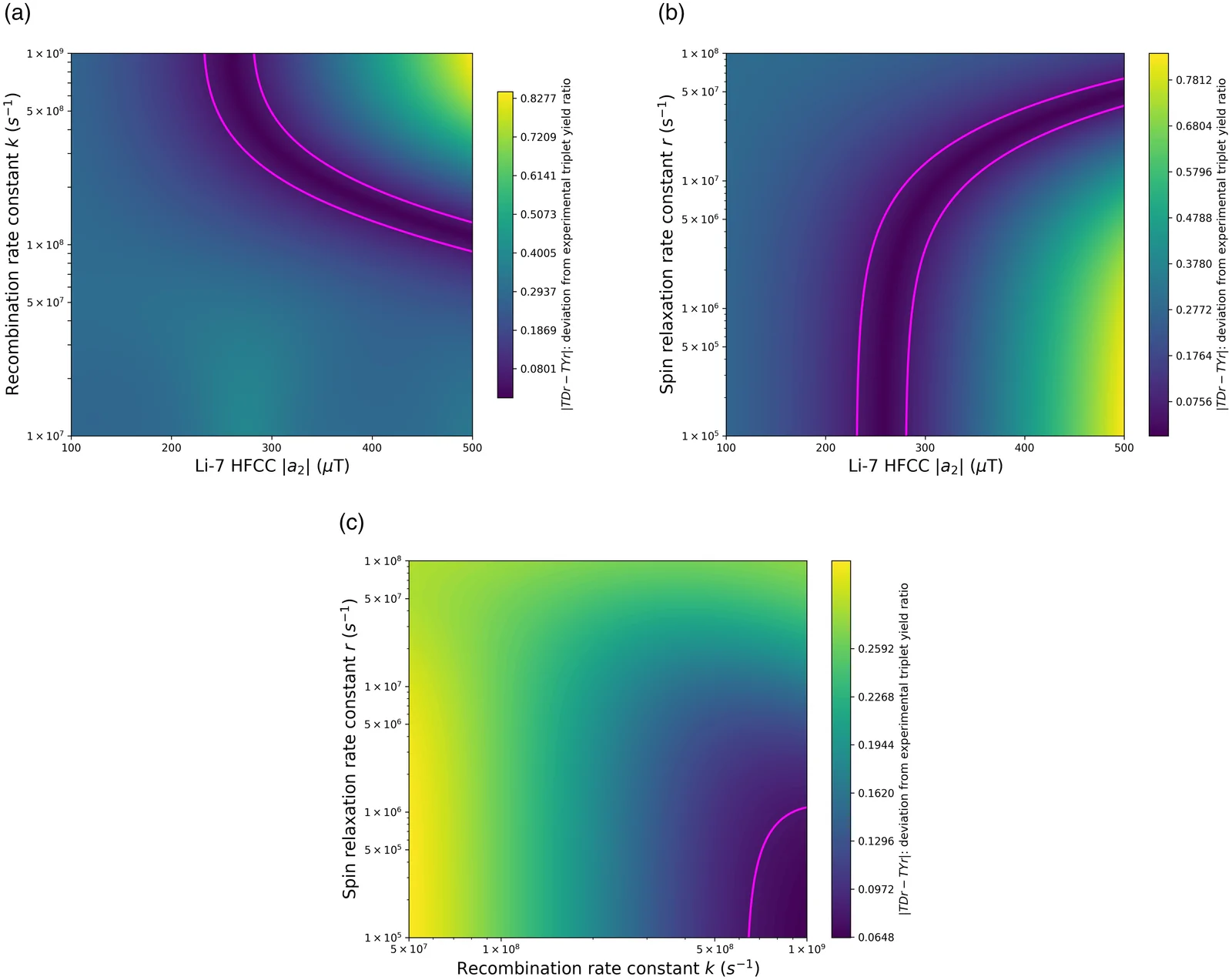
Comparison between theoretical predictions and experimental observations. The absolute difference between radical pair mechanism predictions and experimental data for the triplet yield ratio comparison of ^7^Li versus ^6^Li under varying kinetic conditions. Magenta contour lines indicate regions where theoretical values fall within experimental uncertainty limits. a) Dependence on radical pair reaction rate constant *k* and lithium hyperfine coupling constant |*a*_2_| at fixed spin-coherence relaxation rate $r=1\times 10^{6}\;s^{-1}$, showing theoretical predictions within 6% of experimental values. b) Relationship between spin-coherence relaxation rate *r* and lithium hyperfine coupling constant |*a*_2_| for $k=7.0\times 10^{8}\;s^{-1}$, demonstrating theoretical predictions within 6% of experimental values. c) Correlation between reaction rate *k* and relaxation rate *r* at $|a_{2}|=224\;\mu$T, with theoretical predictions within 8% of experimental values. All analyses performed at B=50μT with both singlet and triplet reaction channels considered.

The RPM model described above was next compared against the behavioural data reported by Ettenberg *et al.* [10], where isotope-dependent effects of lithium were experimentally quantified. We use the Eq. 6 introduced in the Model section as a measure of how closely the RPM matches the experimental isotope ratio. In the heat maps (Fig 5), darker areas indicate where the model and the experiment agree closely (within about 6–8%), while lighter areas indicate larger differences and weaker agreement.

Using this comparison, we tested the ascorbyl mechanism by calculating lithium isotope effects across a wide range of reaction rates. In some regions (still within biologically relevant regions), the model successfully reproduced the strong experimental signals. We then searched for parameter ranges that best matched the experimental data. We found that, despite the biological complexity, the model and experiment agreed well in several regions (|TDr−TYr|≤0.06). This level of agreement was reached across the parameter spaces shown in Fig 5a, 5b, while Fig 5c required a slightly broader tolerance of ≤0.08.

The heat maps in Fig 5 show the absolute difference across three parameter planes under fixed conditions (B=50 μT, $a_{N5}=523.3\;\mu \text{T}$), where *a*_2_ represents the hyperfine coupling constant of ${\;}^{7}Li$ in the ascorbyl radical: (a) the $|a_{2}|-k$ plane, (b) the $|a_{2}|-r$ plane, and (c) the k−r plane.

At fixed conditions (B=50 μT and $a_{N5}=523.3\;\mu$T), the flavin-ascorbyl RP model reproduces experimental results across broad parameter ranges: hyperfine coupling $|a_{2}|\in [210,500]\;\mu$T, reaction rate $k\in [1\times 10^{8},1\times 10^{9}]\;{\text{s}}^{-1}$, and spin relaxation rate $r\in [1\times 10^{6},6\times 10^{7}]\;{\text{s}}^{-1}$. Notably, the predicted hyperfine coupling range aligns well with our DFT calculations for Li^+^-Asc^-^ complexes in both low dielectric environments (ε=2) and aqueous solution. In contrast, assigning H5 as the dominant flavin coupling shifts the magnetic‐field–sensitive range to [234,500] μT (Figure B in Supporting Information S2 Text in S1 File).

## Conclusion

Our primary goal was to determine whether the Radical Pair Mechanism could account for the lithium isotope effects observed in behavioural assays, using a biologically plausible radical-pair alternative to superoxide. Specifically, we examined whether a transient $\left[{FADH}^{•}\cdots {Asc}^{•}\right]$ pair, in the presence of nearby Li^+^, could reproduce the ^7^Li/^6^Li measurements reported by Ettenberg et al. [10].

Notably, ascorbic acid has also been shown to influence brain and behavioural pathways linked to psychiatric conditions like schizophrenia and bipolar disorder [65,66]. Furthermore, recent work has highlighted lithium ascorbate as a potential alternative to conventional lithium carbonate therapy. Chemoreactomic modelling first identified it as a strong candidate, and subsequent cell and animal studies showed improved neuronal survival under stress, higher bioavailability, and lower acute toxicity compared with inorganic lithium salts. All of these results come from the same study. Beyond psychiatric applications, lithium ascorbate shows potential in other areas. A small human study (15 volunteers) further suggests that lithium ascorbate may protect blood proteins and lipids from alcohol-induced oxidative damage, an effect not observed with ascorbic acid alone [67], although the limited sample size prevents firm conclusions. Together, these findings suggest that lithium and ascorbic acid may interact to modulate multiple biological processes. They also raise the possibility that a flavin–ascorbyl-lithium RP is worth considering as a potential theoretical framework to be investigated in the context of lithium’s isotope-dependent effects.

To examine this, we modelled flavin–ascorbyl radical pairs as potential mediators of spin-dependent chemistry. Hyperfine coupling constants for the key radicals were calculated using Density Functional Theory and incorporated into spin-dynamics simulations to predict how lithium isotopes might affect singlet–triplet interconversion. Our results suggest that the nuclear spin of lithium can influence triplet yields, offering a plausible explanation for the behavioural differences observed between ^6^Li and ^7^Li in animal studies [10]. We find triplet-yield ratios of around 1.2, close to the experimental isotope value of 1.3 at Earth-strength magnetic fields, with robust behaviour across different solvent models. Our calculations further indicate that this is possible for certain recombination rate constants, particularly when $k\gg 10^{8}\;{\text{s}}^{-1}$. Moreover, this RP $\left[{FADH}^{•}\cdots {Asc}^{•}\right]$ system could be tested in circadian oscillator models, similar to Zadeh-Haghighi et al., who reported that their superoxide-based RPM model reproduced lithium’s effects in two independent experiments [15].

These findings have two key implications: (i) they provide a potential mechanistic explanation for isotope-specific biological effects, and (ii) they identify flavin–ascorbyl radical pairs as experimentally testable candidates for further study. It is important to note, however, that our model includes several simplifications: (1) we use isotropic hyperfine interactions and omit exchange and dipolar coupling terms, (2) experimental hyperfine constants for lithium ascorbyl are not yet available and (3) the model’s predicted absolute triplet yields are small.

More broadly, one possible interpretation of these results is that lithium may affect redox balance in the brain by altering the spin dynamics of transient flavin–ascorbyl radical pairs (${\text{FADH}}^{•}$–${\text{Asc}}^{•}$) formed during electron-transfer reactions. In such pairs, singlet states allow radicals to recombine into stable products, whereas triplet states keep them separated. A lower triplet yield therefore shortens radical lifetimes by favouring recombination. If ^6^Li shifts the system toward singlet recombination, more radicals would return to the reduced ascorbate form (AscH^-^), which continues to neutralise reactive oxygen species [68]. The ascorbyl radical itself is relatively stable because its unpaired electron is delocalised within the π-system. In contrast, ^7^Li, associated with a higher triplet yield, would allow a larger fraction of radicals to persist, leaving more ascorbate in its less effective radical form (${\text{Asc}}^{•}$) and reducing overall antioxidant capacity [69,70]. Reduced antioxidant activity could increase oxidative stress and may contribute to the stronger hyperactivity observed in rats treated with ^7^Li compared to ^6^Li.

Nevertheless, although a link between oxidative stress and hyperactivity has been tentatively proposed in the context of superoxide-mediated mechanisms [2], and the present findings may be interpreted along similar lines, the connection between the underlying chemistry and behavioural outcomes remains far from established. In this context, the experimental results of Ettenberg et al. [10] serve primarily as a useful comparison, showing that the ascorbic-acid-based model can still reproduce reasonable trends relative to superoxide-based approaches. However, neither model currently explains the mechanistic pathways linking radical-pair chemistry, oxidative imbalance, and behavioural effects such as hyperactivity. Clarifying whether and how these processes converge will require substantial further investigation.

Future experiments could test this idea using the ketamine-induced hyperactivity assay of Ettenberg *et al.* [10], adapted to look for the lithium isotope effects and magnetic sensitivities predicted for the $\left[{FADH}^{•}\cdots {Asc}^{•}\right]$ system. A direct comparison between ^6^Li-Ascorbate and ^7^ Li-Ascorbate-based treatments under controlled magnetic conditions could, in principle, reveal the behavioural signatures suggested by our simulations. Although we predict a stronger effect for ^6^Li–Ascorbate, the actual outcome may depend on biological factors not captured in our model. Moreover, in light of a recent study by Esmaeilpour *et al.* [22] these findings emerge within a broader context that could involve nitric oxide (NO) production. [71–75]. However, further studies are required before any meaningful connection can be established.

Several other radical-pair pathways may be relevant. One example is the $\left[{FAD}^{•-}\cdots {TrpH}^{•+}\right]$ pair proposed by Zadeh-Haghighi et al [15]. It remains an open question whether such TrpH-based systems could also exhibit lithium-dependent behaviour. The radical triad proposed for neurogenesis [25] provides another setting in which lithium isotope effects may be explored. More broadly, identifying additional Li^+^-binding biomolecules capable of forming radical pairs with flavins would help reveal further candidates.

If the RPM contributes even modestly to lithium’s therapeutic action in bipolar disorder, the implications extend beyond this specific drug. Viewing lithium through a spin-chemical lens may help identify general principles for the development of future treatments. For instance, considering isotopic composition or controlled magnetic environments as experimental variables could open new avenues in drug design, enabling more selective modulation of spin-dependent biochemical pathways. Such strategies might also reduce development costs by guiding the search toward compounds that exploit similar spin-chemical features.

In summary, a simple ascorbyl-based radical pair model can, under certain conditions, reproduce the magnitude of reported lithium isotope effects within a biologically realistic RPM model. While the mechanism remains hypothetical, it may be experimentally testable, and its potential role in lithium’s therapeutic effects warrants further investigation.

## Supporting information

S1 FileS1 Text. Benchmark calculations of hyperfine coupling constants (HFCCs) using different DFT functionals.**Table A in S1 Text** lists computed isotropic hyperfine coupling constants for the H4 nucleus of the ascorbyl radical obtained with seven DFT functionals, together with the experimental reference value. **Table B in S1 Text** reports benchmark hyperfine couplings for the Lithium–Ascorbyl radical complex, giving values forH4, ^7^Li and estimated ^6^Li, the latter computed as $a({\;}^{6}Li)=a({\;}^{7}Li)/2.698$. **Fig A in S1 Text** shows the structure of the ${\text{FMNH}}^{•}$ radical, indicating the isoalloxazine ring and the ribityl–phosphate side chain. **Table C in S1 Text** presents computed isotropic HFCCs for the flavin radical (${\text{FMNH}}^{•}$) obtained with three DFT functionals, compared against experimental values. **S2 Text.** Additional calculations using the H5 proton of the flavin radical. **Fig A in S2 Text** displays the magnetic-field dependence of triplet yields in the [FADH(H5) – LiAsc] system, showing the triplet yield ratio (^7^Li/^6^Li) and the absolute triplet yields for each isotope as a function of magnetic field strength. **Fig B in S2 Text** compares theoretical predictions with experimental observations for lithium isotope effects, presenting heat-map panels of the absolute difference between predictions and experimental data under varying kinetic conditions. **S3 Text.** Theoretical framework, including the Hamiltonian formulation, time evolution of the spin density matrix, singlet probability and eigenfrequencies, spin relaxation modelling, and the derivation of the singlet and triplet quantum yields used in the radical pair mechanism calculations. **S4 Text.** Complete Python code for computing the triplet yields of the [${\text{FADH}}^{•}$ – ${\text{Asc}}^{•}$] radical pair with lithium nuclei, using the N5 hyperfine coupling of ${\text{FADH}}^{•}$, including separate implementations of the many-body operators and Hamiltonian for the ^6^Li and ^7^Li isotopes. **S5 Text. Fig C in S5 Text** shows triplet yield ratios under varying kinetic conditions for recombination rates of $4\times 10^{7}$, $4\times 10^{8}$ and $1\times 10^{9}$ s^-1^, illustrating how the triplet yield ratio evolves with increasing recombination rate.(PDF)
