# Supplementary material for: Lithium isotope effects and magnetic interactions in flavin–ascorbyl radical pairs
Source: PLoS One. 2026 Sep 10;21(9):e0356617. doi: 10.1371/journal.pone.0356617 (PMC13561373; doi:10.1371/journal.pone.0356617)
Supplement: Supplementary file 1 [file pone.0356617.s001.pdf]

Supporting information

S1 Text

To determine an appropriate level of theory for hyperfine coupling constant (HFCC) calculations, eight density functional theory (DFT) functionals were benchmarked against the experimental value for the H4 nucleus of the ascorbyl radical. All calculations were carried out in ORCA 6 using identical basis sets and aqueous solvation conditions for a consistent comparison.

Table A. Computed isotropic hyperfine coupling constants ($|H_{12}|$) for the ascorbyl radical using different DFT functionals. All calculations were performed in ORCA 6 with identical basis sets and aqueous solvation conditions.

Functional	Basis set	Solvent	Atom	$|H_{12}|$ (mT)
PWPB95-D4	def2-QZVPP/C	$\varepsilon = 80$	H4	0.1601
RI-B2GP-PLYP-D4	def2-QZVPP/C	$\varepsilon = 80$	H4	0.1551
BP86-D4	def2-QZVPP/C	$\varepsilon = 80$	H4	0.1948
B3LYP-D4	def2-QZVPP/C	$\varepsilon = 80$	H4	0.1349
B3LYP/G-D4	def2-QZVPP/C	$\varepsilon = 80$	H4	0.1346
TPSS-D4	def2-QZVPP/C	$\varepsilon = 80$	H4	0.1798
M06-D4	def2-QZVPP/C	$\varepsilon = 80$	H4	0.1769
Experimental	—	—	H4	1.1760

The experimental reference values was extracted from Laroff et al [1]. In Table B, we used aqueous conditions for the free ascorbyl radical to facilitate comparison with the experiment.

For the Lithium-Ascorbyl complex, we switched to $\varepsilon = 2$ to match the conditions used for superoxide in our RPM calculations. Running LiAsc in water only slightly lowers the H and ^{7}Li couplings and does not change the overall triplet yield, so using $\varepsilon = 2$ keeps the comparison consistent. The geometry of the Lithium-Ascorbyl complex was optimised at the B3LYP-D4/6-311++G(d,p) level of theory.

Table B. Benchmark hyperfine couplings for the Lithium-Ascorbyl radical complex. Values are reported in mT for ^{12}H, ^{7}Li, and estimated ^{6}Li (computed as $a(^6\text{Li}) = a(^7\text{Li})/2.698$).

Functional	Basis set	Solvent	^{12}H (mT)	^{7}Li (mT)	^{6}Li (mT)
PWPB95-D4	def2-QZVPP	eps(2)	0.3980	-0.1583	-0.0587
RI-B2GP-PLYP-D4	def2-QZVPP	eps(2)	0.3954	-0.1451	-0.0538
BP86-D4	def2-QZVPP	eps(2)	0.4205	-0.1154	-0.0428
B3LYP/G-D4	def2-QZVPP	eps(2)	0.3783	-0.1466	-0.0543
B3LYP-D4	def2-QZVPP	eps(2)	0.3777	-0.1483	-0.0550
TPSS-D4	def2-QZVPP	eps(2)	0.4157	-0.1256	-0.0466
M06-D4	def2-QZVPP	eps(2)	0.4229	-0.2239	-0.0830

Fig A. Structure of the FMNH$^\bullet$ radical. The isoalloxazine ring (bottom) forms the flavin semiquinone and carries the unpaired electron. The circled region shows the ribityl–phosphate side chain. We retain the full structure here for comparison with experimental FADH$^\bullet$, but the hyperfine calculations were performed on the flavin semiquinone only, since the tail does not significantly contribute to the spin density in our RP model.

To further validate the method, hyperfine coupling constants (HFCCs) were calculated for the flavin radical ($FMNH^{\bullet}$) using several DFT functionals. Geometry optimizations were performed at the B3LYP-D3/6-311++G(d,p) level with $\varepsilon = 2$. The resulting isotropic HFCCs are presented in Table C.

Table C. Computed isotropic hyperfine coupling constants (a) for flavin ($FMNH^{\bullet}$) obtained using different DFT functionals. Geometry optimization was performed at the B3LYP-D3/6-311++G(d,p) level with $\varepsilon = 2$.

Functional	Basis set	Solvent	Atom (ASE)	Atom (lit.)	a (MHz)	a (mT)	Exp. (mT)
**M06-D4**	def2-QZVPP/C	$\varepsilon = 2$	N8	N5	16.9969	0.6065	0.5167
			N25	N10	9.0168	0.3217	0.3115
			H50	H5	22.9054	0.8173	0.8043
**TPSS-D4**	def2-QZVPP/C	$\varepsilon = 2$	N8	N5	13.0905	0.4671	0.5167
			N25	N10	8.2294	0.2936	0.3115
			H50	H5	-19.3599	-0.6908	0.8043
**revTPSS-D4**	def2-QZVPP/C	$\varepsilon = 2$	N8	N5	16.8294	0.6005	0.5167
			N25	N10	10.0641	0.3591	0.3115
			H50	H5	-20.0912	-0.7169	0.8043

S2 Text

For completeness, we also report calculations using the H5 proton, which has the largest hyperfine coupling in the flavin radical. Because a_{H5} exceeds the N5 coupling, H5 could act as the dominant contribution to the spin dynamics. The figures below show that the overall lithium-isotope behaviour remains consistent with the N5 results, with shifts in absolute yields and in the ^{7}Li/^{6}Li ratio reflecting the stronger H5 coupling.

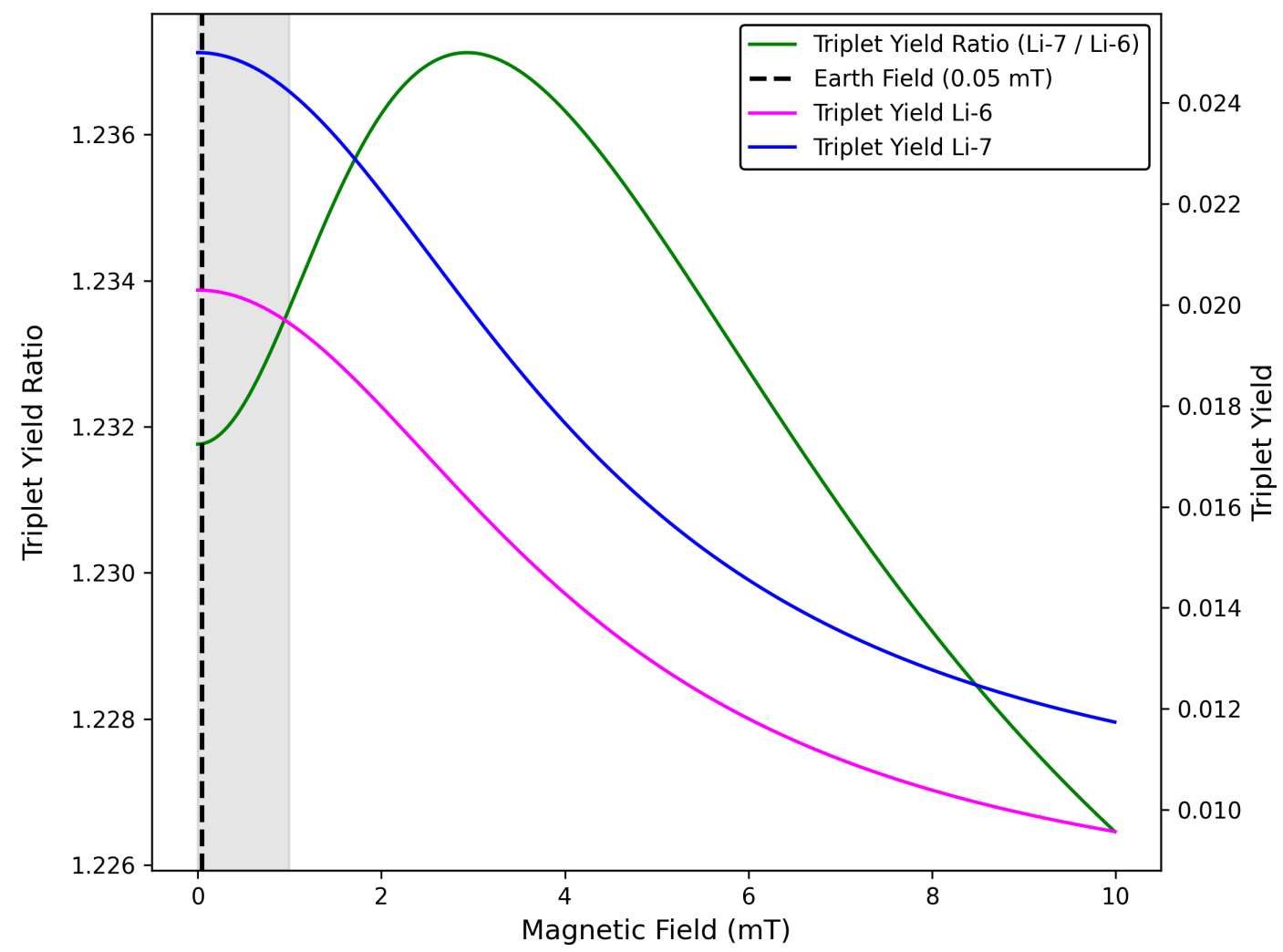

Fig A. Magnetic-field dependence of triplet yields in the [FADH(H5) − LiAsc] system. The green solid line denotes the triplet yield ratio (^{7}Li/^{6}Li), while the magenta and blue curves represent the absolute triplet yields for ^{6}Li and ^{7}Li, respectively, as a function of the applied magnetic field strength. The vertical dashed black line indicates the Earth's magnetic field (0.05 mT), and the grey shaded region highlights the low-field regime. The hyperfine coupling parameters are: $a_{H5} = 0.803\,mT$, $a_{Li6} = 0.083\,mT$, $a_{Li7} = 0.224\,mT$, and $a_{H} = 0.423\,mT$ (corresponding to the dominant hydrogen of the ascorbyl radical). Reaction and relaxation rates are $k = 7 \times 10^{8}\,s^{-1}$ and $r = 10^{6}\,s^{-1}$, respectively.

Moreover, to evaluate how the dominant H5 coupling influences the kinetic parameter space, we also computed the corresponding reaction-rate and relaxation-rate dependencies. These results are summarized in the heat-map panels of Fig. B.

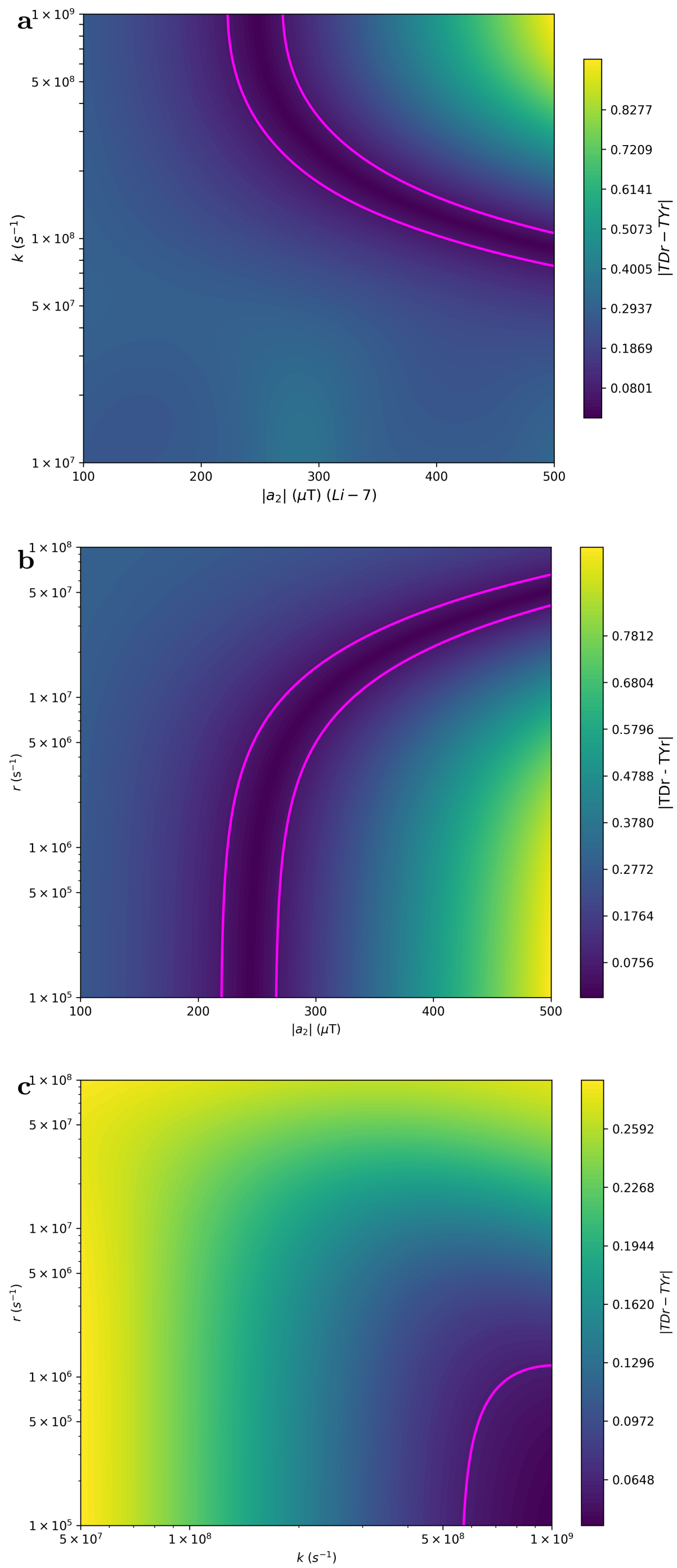

Fig B. Comparison between theoretical predictions and experimental observations for lithium isotope effects. The absolute difference between radical pair mechanism predictions and experimental data for the triplet yield ratio comparison of ^{7}Li versus ^{6}Li under varying kinetic conditions. Magenta contour lines indicate regions where theoretical values fall within experimental uncertainty limits. **a)** Dependence on radical pair reaction rate constant k and lithium hyperfine coupling constant $|a_2|$ at fixed spin-coherence relaxation rate $r = 1 \times 10^6\,\mathrm{s}^{-1}$, showing theoretical predictions within 6% of experimental values. **b)** Relationship between spin-coherence relaxation rate r and lithium hyperfine coupling constant $|a_2|$ for $k = 7.0 \times 10^8\,\mathrm{s}^{-1}$, demonstrating theoretical predictions within 6% of experimental values. **c)** Correlation between reaction rate k and relaxation rate r at $|a_2| = 0.224\,\mu\mathrm{T}$, with theoretical predictions within 6% of experimental values. All analyses performed at $B = 50\,\mu\mathrm{T}$ with both singlet and triplet reaction channels considered.

S3 Text

Hamiltonian and Time Evolution. To determine magnetic field effects (MFEs), the singlet character of the pair can be calculated via the singlet projection operator, leading to the following initial density matrix:

$$\hat{\rho}(0) = \frac{1}{M}\hat{P}^S, \tag{1}$$

where the singlet projection operator is defined as:

$$\hat{P}^S = |S\rangle\langle S| \otimes \hat{1}_M, \tag{2}$$

and $\hat{1}_M$ is the identity operator in the nuclear spin subspace, while $\frac{1}{M}$ provides normalization over the number of nuclear spin states, with:

$$M = \prod_j (2I_j + 1), \tag{3}$$

where I_j is the spin quantum number for the jth nucleus, and the product is taken over all nuclei in the radical.

The initial singlet radical pair evolves over time under the spin Hamiltonian $\hat{H}$, primarily influenced by external magnetic fields (Zeeman interaction) and the coupling between electron and nuclear spins (hyperfine interactions). For simplicity, we focus on these dominant effects, excluding exchange and dipolar couplings:

$$\hat{H} = \omega \hat{S}_{A_z} + \hat{\mathbf{S}}_A \cdot \sum_{i=1}^{N_A} a_{A_i} \hat{\mathbf{I}}_{A_i} + \omega \hat{S}_{D_z} + \hat{\mathbf{S}}_D \cdot \sum_{i=1}^{N_D} a_{D_i} \hat{\mathbf{I}}_{D_i}. \tag{4}$$

We use the following equation to describe the spin state of a radical. For computational models, the electron spin vector operator is typically represented using matrices in Hilbert space, expressed as.

$$\hat{S} = \begin{pmatrix} 0 & 1/2 \\ 1/2 & 0 \end{pmatrix} \mathbf{i} + \begin{pmatrix} 0 & -i/2 \\ i/2 & 0 \end{pmatrix} \mathbf{j} + \begin{pmatrix} 1/2 & 0 \\ 0 & -1/2 \end{pmatrix} \mathbf{k} \tag{5}$$

Where i, j, k are mutually orthogonal unit vectors. Similar matrices can be constructed for other spin particles with any S (or I).

Here, $\hat{\mathbf{S}}_A$ and $\hat{\mathbf{S}}_D$ are the spin operators for the unpaired electrons in radicals $A^{\cdot-}$ (acceptor) and $D^{\cdot+}$ (donor), respectively. Similarly, $\hat{\mathbf{I}}_A$ and $\hat{\mathbf{I}}_D$ represent the nuclear spin operators for the acceptor and donor radical molecules. The isotropic hyperfine coupling constants are denoted by a_A and a_D, while N_A and N_D indicate the number of nuclei coupled to electrons A and D, respectively. Finally, $\omega = \frac{g\mu_B B}{\hbar}$ is the electron Larmor frequency due to the external magnetic field B.

The system is described by a spin density matrix, $\rho(t)$, whose time evolution is governed by the spin Hamiltonian (Equation 3.6). Using the Liouville–Von Neumann equation, the dynamics are expressed as:

$$\frac{d\hat{\rho}}{dt} = -i[\hat{H}, \hat{\rho}]. \tag{6}$$

Assuming a time-independent Hamiltonian $\hat{H}$, the formal solution to eq (Equation 3.15) is:

$$\hat{\rho}(t) = e^{-i\hat{H}t}\hat{\rho}(0)e^{i\hat{H}t} = \frac{1}{M}e^{-i\hat{H}t}\hat{P}^S e^{i\hat{H}t} \tag{7}$$

Singlet Probability and Eigenfrequencies. Having the spin density matrix from Eq. (3.11), the probability of finding a singlet state can be calculated as the trace of the projection operator. This procedure requires inserting the identity operator expressed in terms of the eigenstates of the Hamiltonian, $\hat{I} = \sum_{n=1}^{M} |n\rangle\langle n|$.

$$\rho_S(t) = \mathrm{Tr}[\hat{P}_S\hat{\rho}(t)] = \sum_{m=1}^{4M}\langle m|\hat{P}_S\hat{\rho}(t)|m\rangle = \sum_{m=1}^{4M}\sum_{n=1}^{4M}\langle m|\hat{\rho}(t)|n\rangle\langle n|\hat{P}_S|m\rangle \tag{8}$$

Here, the trace runs over all $4M$ states of the Hilbert space because they are four spin states for the electrons and M nuclear states. The eigenstates $|m\rangle$ and $|n\rangle$ of $\hat{H}$ correspond to the energies given by:

$$\omega_m = \langle m|\hat{H}|m\rangle \quad \text{and} \quad \omega_n = \langle n|\hat{H}|n\rangle.$$

Here, ω_m and ω_n are the total eigenfrequencies from the full Hamiltonian (including hyperfine and Zeeman terms), whereas $\omega = \gamma B_0$ is specifically the Larmor precession frequency due to the external field.

$$\omega_{mn} = \omega_m - \omega_n \tag{9}$$

Because the matrix representation of $\hat{H}$ has dimension $4M \times 4M$ (four electron-spin states times the M nuclear spin states), $\rho_S(t)$ emerges as a sum of diagonal (population) terms and off-diagonal (coherence) terms in the eigenbasis. The off-diagonal elements typically evolve in time as $\exp(\pm i\,\omega_{mn}\,t)$, where $\omega_{mn} = \omega_m - \omega_n$. These coherence terms are important for spin-state interconversion.

We apply the cyclic invariance property of the trace ($\mathrm{Tr}(ABC) = \mathrm{Tr}(BCA) = \mathrm{Tr}(CAB)$) and the Hermiticity of $\hat{P}$ to establish the equality in the following equation.

Considering that the matrix element $\langle m|\hat{\rho}(t)|n\rangle$ can be expressed as $\rho_{nm}(t)$, we derive its components using eq (3.12) as follows:

$$\rho_{nm}(t) = \frac{1}{M}\langle n|e^{-i\hat{H}t}|n\rangle\langle n|\hat{P}^S|m\rangle\langle m|e^{+i\hat{H}t}|m\rangle = \frac{1}{M}P^S_{nm}\exp(i\omega_{mn}t) \tag{10}$$

where $\omega_{mn} = E_m - E_n$, with E_α representing the energy of the state α. So then $\rho_S(t)$ becomes:

$$\rho_S(t) = \sum_{m=1}^{4M}\sum_{n=1}^{4M}\left|\langle n|\hat{P}^S|m\rangle^2\right|\cos(\omega_m - \omega_n)t \tag{11}$$

Spin Relaxation. Spin relaxation, which causes loss of coherence, is often modeled by an exponential decay (rate r) to capture key effects without complicating the Hamiltonian. Following Bagryansky et al. [2], we introduce relaxation phenomenologically as:

$$\rho_S(t) = \frac{1}{4} - \left(\rho_S - \frac{1}{4}\right)e^{-rt} = p(t) \tag{12}$$

Quantum Yields and Reencounter Probability. When multiple reaction pathways are possible, it is often important to identify the fraction of radical pairs that follow each route, a quantity known as the *quantum yield*. However, for a diffusing radical pair (RP), spin-state mixing slows at short distances where strong interactions (exchange, dipolar) dominate. In simple models, mixing occurs freely when the radicals are far apart but stops within a defined "encounter distance." Consequently, the final

singlet yield must include the *reencounter probability* $f(t)$, frequently modeled as $f(t) = k\,e^{-kt}$. The rate constant k represents the first-order recombination rate constant for radical pairs. Thus, the final singlet yield for periods larger than the radical pair lifetime:

$$\Phi_S = k \int_0^\infty p(t) e^{-kt}\,dt, \tag{13}$$

The final singlet yield from Eq. (C13) is obtained by substituting the expression for $p(t)$ given in Eq. (C12):

$$\Phi_S = k \int_0^\infty \left[\frac{1}{4} - \left(\frac{1}{4} - \rho_S(t)\right) e^{-rt}\right] e^{-kt}\; dt \tag{14}$$

Expanding the integral in a sum of three terms:

$$\Phi_S = \frac{1}{4}\int_0^\infty k e^{-kt}\,dt - \int_0^\infty \frac{k}{4} e^{-rt} e^{-kt}\,dt + \mathbf{k}\int_{\mathbf{0}}^\infty \langle \mathbf{n}|\hat{\mathbf{P}}^{\mathbf{S}}|\mathbf{m}\rangle^{\mathbf{2}} \cos(\omega_{\mathbf{m}} - \omega_{\mathbf{n}})\mathbf{t}\mathbf{e}^{-\mathbf{kt}}\mathbf{e}^{-\mathbf{rt}}\;\mathbf{dt} \tag{15}$$

To solve the third part of the integral, we use the Fourier transform:

$$\int_0^\infty \cos(\omega_{mn} t) e^{-\alpha t}\,dt = \frac{\alpha}{\alpha^2 + \omega_{mn}^2} \tag{16}$$

where $\alpha = k + r$. Solving the integral gives us:

$$\Phi_S = \frac{1}{4}\cdot k \cdot \frac{1}{k} - \frac{k}{4}\cdot\frac{1}{k+r} + k\sum_{m=1}^{4M}\sum_{n=1}^{4M} \frac{(k+r)}{(k+r)^2 + (\omega_m - \omega_n)^2}\left|\langle n|\hat{P}^S|m\rangle\right|^2 \tag{17}$$

Final singlet yield:

$$\Phi_S = \frac{1}{4} - \frac{k}{4(k+r)} + \frac{1}{M}\sum_{m=1}^{4M}\sum_{n=1}^{4M} \frac{(k+r)}{(k+r)^2 + (\omega_m - \omega_n)^2}\left|\langle n|\hat{P}^S|m\rangle\right|^2 \tag{18}$$

The triplet yield is given by:

$$\Phi_T = 1 - \Phi_S \tag{19}$$

$$\Phi_S = \frac{1}{4} - \frac{k}{4(k+r)} + \frac{1}{M}\sum_{m=1}^{4M}\sum_{n=1}^{4M} \frac{(k+r)}{(k+r)^2 + (\omega_m - \omega_n)^2}\left|\langle n|\hat{P}^S|m\rangle\right|^2. \tag{20}$$

The triplet yield is given by:

$$\Phi_T = 1 - \Phi_S \tag{21}$$

Both Φ_S and Φ_T are dimensionless yields corresponding to the fraction of radical pairs recombining through the singlet or triplet pathway, respectively. Substituting the expression for the singlet yield, the triplet yield [3] becomes:

$$\Phi_T = \frac{3(k+r)+k}{4(k+r)} - \frac{1}{M}\sum_{m,n=1}^{4M} \frac{\left|r\langle m|\hat{\rho}^S|n\rangle\right|^2 k(k+r)}{(k+r)^2 + (\omega_m - \omega_n)^2} \tag{22}$$

Note that the largest contribution to the quantum yield is obtained for a radical pair with a close-packed spectrum of eigenvalues, and degenerate eigenstates potentially contribute the most.

S4 Text

1 Python code for N5

This appendix contains the complete Python script used to compute the triplet yields of the [FADH$^{\bullet} \cdots$ Asc$^{\bullet}$] radical pair in the presence of nearby lithium nuclei. The hyperfine interaction included here corresponds specifically to the *N5 nitrogen nucleus* of FADH$^{\bullet}$. A similar code was used for the **H5 hydrogen nucleus**, where the nuclear spin changes from 1 (nitrogen) to 1/2 (hydrogen), requiring only the substitution of the appropriate spin matrices. The code evaluates the isotope dependence of ^{6}Li and ^{7}Li across a range of magnetic field strengths.

- **A1.** Imports and helper functions
- **A2.** Physical constants
- **A3.** Spin matrices
- **A4.** Construction of many-body operators for Li-6
- **A5.** Hamiltonian and triplet yield for Li-6
- **A6.** Construction of many-body operators for Li-7
- **A7.** Hamiltonian and triplet yield for Li-7
- **A8.** Triplet Yield Ratio Function
- **A7.** Simulation and plotting

1.1 Python script

```
 ============================================================
 A1. IMPORTS AND BASIC UTILITIES
 ============================================================
import numpy as np
import matplotlib.pyplot as plt
from scipy.linalg import eigh
from functools import reduce
import seaborn as sns

# Tensor product helper
def kron(oplist):
    """Compute Kronecker product of a list of operators."""
    return reduce(np.kron, oplist)

 ============================================================
 A2. CONSTANTS AND PHYSICAL PARAMETERS
 ============================================================
gamma_e = 1.76086e8 #Electron gyromagnetic ratio (rad/(s*mT)) e

# Hyperfine coupling constants (mT  e  rad/s)
hfc_Ni = 0.606 * gamma_e # FADH radical nitrogen (N5) (spin 1)
hfc_Li6 = -0.083 * gamma_e # Li-6 hyperfine (spin 1)
hfc_Li7 = -0.224 * gamma_e # Li-7 hyperfine (spin 3/2)
hfc_H = 0.423 * gamma_e # Ascorbyl proton (spin 1/2)

```

```
# Kinetic rates
k = 7e8 # Recombination rate
r = 1e6 # Relaxation rate

# Imaginary unit
j = 1j
 ============================================================
 A3. SPIN MATRICES
 ============================================================

# Spin-1/2 Pauli matrices (for electrons and hydrogen)
sigma_x = 0.5 * np.array([[0, 1],
                          [1, 0]])
sigma_y = 0.5 * np.array([[0, -1j],
                          [1j, 0]])
sigma_z = 0.5 * np.array([[1, 0],
                          [0, -1]])

# Spin-1 matrices (nitrogen, Li-6 case)
S1_x = (1 / np.sqrt(2)) * np.array([[0, 1, 0],
                                    [1, 0, 1],
                                    [0, 1, 0]])
S1_y = (1 / np.sqrt(2)) * np.array([[0, -j, 0],
                                    [j, 0, -j],
                                    [0, j, 0]])
S1_z = np.array([[1, 0, 0],
                 [0, 0, 0],
                 [0, 0, -1]])

# Spin-3/2 matrices (Li-7 nuclear spin)
I7_x = 0.5 * np.array([[0, np.sqrt(3), 0, 0],
                       [np.sqrt(3), 0, 2, 0],
                       [0, 2, 0, np.sqrt(3)],
                       [0, 0, np.sqrt(3), 0]])
I7_y = 0.5 * np.array([[0, -j * np.sqrt(3), 0, 0],
                       [j * np.sqrt(3), 0, -2 * j, 0],
                       [0, 2 * j, 0, -j * np.sqrt(3)],
                       [0, 0, j * np.sqrt(3), 0]])
I7_z = 0.5 * np.array([[3, 0, 0, 0],
                       [0, 1, 0, 0],
                       [0, 0, -1, 0],
                       [0, 0, 0, -3]])

# Identity matrices
I_2 = np.eye(2) # spin-1/2
I_3 = np.eye(3) # spin-1
I_4 = np.eye(4) # spin-3/2
 ============================================================
 A4. MANY-BODY OPERATORS (Li-6 SYSTEM)
 Ordering: [eA, eB, N, Li-6, H] = [2,2,3,3,2]
 ============================================================

# Electron A
S_Ax = kron([sigma_x, I_2, I_3, I_3, I_2])
S_Ay = kron([sigma_y, I_2, I_3, I_3, I_2])
S_Az = kron([sigma_z, I_2, I_3, I_3, I_2])

```

```
# Electron B
S_Bx = kron([I_2, sigma_x, I_3, I_3, I_2])
S_By = kron([I_2, sigma_y, I_3, I_3, I_2])
S_Bz = kron([I_2, sigma_z, I_3, I_3, I_2])

# Nitrogen (spin-1)
I_Nx = kron([I_2, I_2, S1_x, I_3, I_2])
I_Ny = kron([I_2, I_2, S1_y, I_3, I_2])
I_Nz = kron([I_2, I_2, S1_z, I_3, I_2])

# Lithium-6 (spin-1)
I_Lx6 = kron([I_2, I_2, I_3, S1_x, I_2])
I_Ly6 = kron([I_2, I_2, I_3, S1_y, I_2])
I_Lz6 = kron([I_2, I_2, I_3, S1_z, I_2])

# Hydrogen
I_Hx = kron([I_2, I_2, I_3, I_3, sigma_x])
I_Hy = kron([I_2, I_2, I_3, I_3, sigma_y])
I_Hz = kron([I_2, I_2, I_3, I_3, sigma_z])
 ============================================================
 A5. HAMILTONIAN AND TRIPLET YIELD  Li-6 CASE
 ============================================================

def TripletYield_Li6_with_H(B, k, r):

    omega = -gamma_e * B
    dim = 2 * 2 * 3 * 3 * 2 # = 72

    # Total spin Hamiltonian
    H = (
        hfc_Ni * (S_Ax @ I_Nx + S_Ay @ I_Ny + S_Az @ I_Nz)
        + hfc_Li6 * (S_Bx @ I_Lx6 + S_By @ I_Ly6 + S_Bz @ I_Lz6)
        + hfc_H * (S_Bx @ I_Hx + S_By @ I_Hy + S_Bz @ I_Hz)
        + omega * (S_Az + S_Bz)
    )

    # Diagonalize Hamiltonian
    e, v = eigh(H)

    # Singlet projector (electron subspace)
    PS_e = (
        0.25 * np.eye(4)
        - (np.kron(sigma_x, sigma_x)
           + np.kron(sigma_y, sigma_y)
           + np.kron(sigma_z, sigma_z))
    )

    # Embed projector into full Hilbert space
    PS = kron([PS_e, I_3, I_3, I_2])
    PS = np.conj(v.T) @ PS @ v

    # Triplet yield formula
    Phi_T = (
        (3 * (k + r) + k) / (4 * (k + r))
        - (1 / 18)
        * sum(
            sum(
```

```python
                (np.abs(PS[m, n]) ** 2 * k * (k + r))
                / ((k + r)**2 + (e[m] - e[n])**2)
                for n in range(dim)
            )
            for m in range(dim)
        )
    )

    return Phi_T.real

 ============================================================
 A6. MANY-BODY OPERATORS (Li-7 SYSTEM)
 Ordering: [eA, eB, N, Li-7, H] = [2,2,3,4,2]
 ============================================================

# Electron A
S_Ax_Li7 = kron([sigma_x, I_2, I_3, I_4, I_2])
S_Ay_Li7 = kron([sigma_y, I_2, I_3, I_4, I_2])
S_Az_Li7 = kron([sigma_z, I_2, I_3, I_4, I_2])

# Electron B
S_Bx_Li7 = kron([I_2, sigma_x, I_3, I_4, I_2])
S_By_Li7 = kron([I_2, sigma_y, I_3, I_4, I_2])
S_Bz_Li7 = kron([I_2, sigma_z, I_3, I_4, I_2])

# Nitrogen
I_Nx_Li7 = kron([I_2, I_2, S1_x, I_4, I_2])
I_Ny_Li7 = kron([I_2, I_2, S1_y, I_4, I_2])
I_Nz_Li7 = kron([I_2, I_2, S1_z, I_4, I_2])

# Lithium-7 (spin-3/2)
I_Lx7 = kron([I_2, I_2, I_3, I7_x, I_2])
I_Ly7 = kron([I_2, I_2, I_3, I7_y, I_2])
I_Lz7 = kron([I_2, I_2, I_3, I7_z, I_2])

# Hydrogen
I_Hx_Li7 = kron([I_2, I_2, I_3, I_4, sigma_x])
I_Hy_Li7 = kron([I_2, I_2, I_3, I_4, sigma_y])
I_Hz_Li7 = kron([I_2, I_2, I_3, I_4, sigma_z])

============================================================
 A7. HAMILTONIAN AND TRIPLET YIELD  Li-7 CASE
 ============================================================

def TripletYield_Li7_with_H(B, k, r):

    omega = -gamma_e * B
    dim = 2 * 2 * 3 * 4 * 2 # = 96

    H = (
        hfc_Ni * (S_Ax_Li7 @ I_Nx_Li7 + S_Ay_Li7 @ I_Ny_Li7 + S_Az_Li7 @ I_Nz_Li7)
        + hfc_Li7 * (S_Bx_Li7 @ I_Lx7 + S_By_Li7 @ I_Ly7 + S_Bz_Li7 @ I_Lz7)
        + hfc_H * (S_Bx_Li7 @ I_Hx_Li7 + S_By_Li7 @ I_Hy_Li7 + S_Bz_Li7 @ I_Hz_Li7)
        + omega * (S_Az_Li7 + S_Bz_Li7)
    )
```

```
    e, v = eigh(H)

    PS_e = (
        0.25 * np.eye(4)
        - (np.kron(sigma_x, sigma_x)
           + np.kron(sigma_y, sigma_y)
           + np.kron(sigma_z, sigma_z))
    )

    PS = kron([PS_e, np.eye(3), np.eye(4), I_2])
    PS = np.conj(v.T) @ PS @ v

    Phi_T = (
        (3 * (k + r) + k) / (4 * (k + r))
        - (1 / 24)
        * sum(
            sum(
                (np.abs(PS[m, n]) ** 2 * k * (k + r))
                / ((k + r)**2 + (e[m] - e[n])**2)
                for n in range(dim)
            )
            for m in range(dim)
        )
    )

    return Phi_T.real
 ============================================================
 A8. RATIO FUNCTION
 ============================================================

def TripletYieldRatio(B, k, r):
    """Return _T(Li-7) / _T(Li-6)."""
    return TripletYield_Li7_with_H(B, k, r) / TripletYield_Li6_with_H(B, k, r)
 ============================================================
 A9. EXAMPLE SIMULATION AND PLOTTING
 ============================================================

Bmin, Bmax, Bstep = 0.0, 10.0, 0.01
B_values = np.arange(Bmin, Bmax, Bstep)

# Compute yields
triplet_yield_Li6_new = [TripletYield_Li6_with_H(B, k, r) for B in B_values]
triplet_yield_Li7_new = [TripletYield_Li7_with_H(B, k, r) for B in B_values]
triplet_yield_ratio = [TripletYieldRatio(B, k, r) for B in B_values]

# Figure
plt.figure(figsize=(8, 6))
ax1 = plt.gca()
ax1.plot(B_values, triplet_yield_ratio, 'g-', label='Triplet Yield Ratio (Li-7 / Li-6)')
ax1.set_xlabel('Magnetic Field (mT)', fontsize=12)
ax1.set_ylabel('Triplet Yield Ratio', fontsize=12, color='black')
ax1.tick_params(axis='y', labelcolor='black')
ax2 = ax1.twinx()
```

```
ax2.plot(B_values, triplet_yield_Li6_new, ’magenta’, label=’Triplet Yield Li-6’)
ax2.plot(B_values, triplet_yield_Li7_new, ’blue’, label=’Triplet Yield Li-7’)
ax2.set_ylabel(’Triplet Yield’, fontsize=12)
ax1.axvspan(0.0, 1.0, color=’grey’, alpha=0.2)
ax1.axvline(x=0.05, color=’black’, linestyle=’--’, linewidth=2,
           label="Earth Field (0.05 mT)")
lines1, labels1 = ax1.get_legend_handles_labels()
lines2, labels2 = ax2.get_legend_handles_labels()
ax1.legend(lines1 + lines2, labels1 + labels2, loc=’upper right’)
B_earth = 0.05
li6_earth = TripletYield_Li6_with_H(B_earth, k, r)
li7_earth = TripletYield_Li7_with_H(B_earth, k, r)
ratio_earth = li7_earth / li6_earth

print(f" Ratio (Li-7 / Li-6) = {ratio_earth:.5f}")

plt.tight_layout()
plt.savefig(’Lithium_Ascorbyl_N5_ext_B.png’, dpi=300, bbox_inches=’tight’)
plt.show()
```

S5 Text

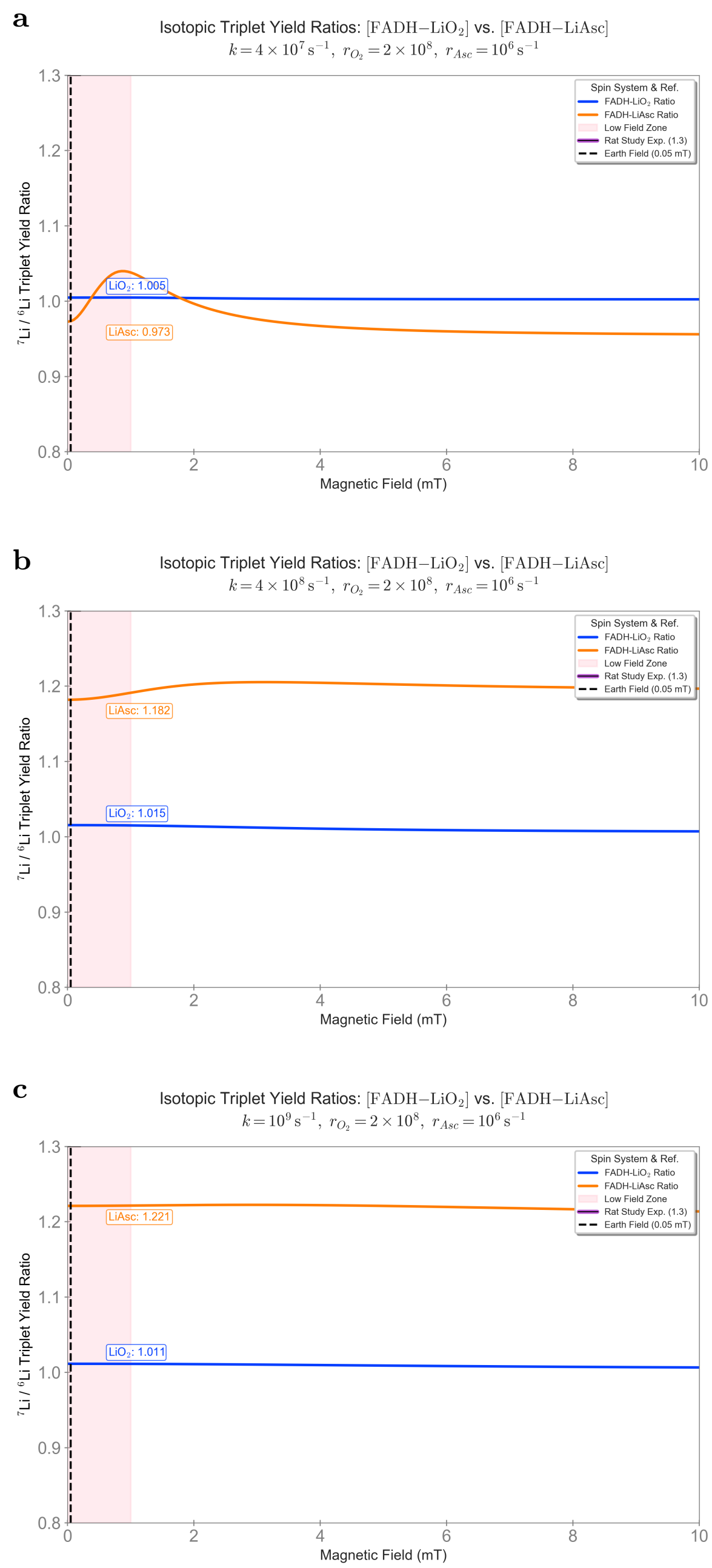

Fig C. Triplet yield ratios under varying kinetic conditions. a, $k = 4 \times 10^7\,\mathrm{s}^{-1}$. **b,** $k = 4 \times 10^8\,\mathrm{s}^{-1}$. **c,** $k = 1 \times 10^9\,\mathrm{s}^{-1}$. Each panel illustrates how the triplet yield ratio evolves with increasing recombination rates. For realistic relaxation times, ascorbyl has $\tau \approx 1\,\mathrm{ms}$ ($r \approx 10^6\,\mathrm{s}^{-1}$), and superoxide has $\tau \approx 4.9\,\mathrm{ns}$ ($r \approx 2 \times 10^8\,\mathrm{s}^{-1}$).

References

1. Lagercrantz NYC, Kwan T. The Electron Spin Resonance Spectra of Radical Intermediates in the Oxidation of Ascorbic Acid and Related Substances'; 1961. 4.

Available from: https://pubs.acs.org/sharingguidelines.

2. Bagryansky VA, Borovkov VI, Molin YN. Quantum beats in radical pairs. Russian Chemical Reviews. 2007 6;76(6):493-506. doi:10.1070/rc2007v076n06abeh003715.

3. Hore PJ. Upper bound on the biological effects of 50/60 Hz magnetic fields mediated by radical pairs. 2019. Available from: https://doi.org/10.7554/eLife.44179.001. doi:10.7554/eLife.44179.001.
